# Selective SIK2/SIK3 inhibition reprograms pro- and antiinflammatory pathways in myeloid cells, improving autoimmune disease outcomes

**DOI:** 10.1172/jci.insight.171776

**Published:** 2026-02-09

**Authors:** Steve De Vos, Nicolas Desroy, Susan J. Bellaire, Anna Pereira Fernandes, Stéphanie Lavazais, Didier Merciris, Carole Delachaume, Catherine Robin-Jagerschmidt, Adrien Cosson, Angela Lazaryan, Nancy Van Osselaer, David Amantini, Christophe Peixoto, Maikel L. Colli, Thomas Van Eeckhoutte, Tiina Hakonen, Magali Constant, Alberto Garcia-Hernandez, Rahul Barron, Geert D’Haens, Wulf O. Böcher

**Affiliations:** 1 Galapagos NV, Mechelen, Belgium.; 2 Galapagos SASU, Romainville, France.; 3 Galapagos BV, Leiden, Netherlands.; 4 Galapagos GmbH, Basel, Switzerland.; 5 Department of Gastroenterology and Hepatology, Amsterdam University Medical Centres, Amsterdam, Netherlands.

**Keywords:** Autoimmunity, Dermatology, Gastroenterology, Autoimmune diseases, Clinical trials, Mouse models

## Abstract

Adaptive immune responses are widely considered the primary drivers of chronic inflammation in autoimmune disease, yet increasing evidence suggests that dysregulated myeloid cells play a central role in sustaining tissue damage. Salt-inducible kinases (SIKs) regulate immune cell activation, and their pharmacological inhibition can promote a shift from proinflammatory toward an immunoregulatory phenotype. We investigated whether selective inhibition of SIK2 and SIK3 with GLPG3970 could reprogram monocytes, macrophages, and dendritic cells, and we assessed pharmacological effects on activated T and B cells. Preclinical studies in mouse models of colitis, psoriasis, and arthritis demonstrated that SIK2/SIK3 inhibition reduced inflammatory activity and promoted immunoregulatory and tolerogenic-associated pathways. Clinical signal-detection studies in ulcerative colitis, psoriasis, and rheumatoid arthritis revealed signs of clinical and biological activity in ulcerative colitis and psoriasis. These findings suggest that myeloid cell dysfunction and impaired myeloid phenotype switching contribute to chronic inflammation in autoimmune diseases and that therapeutic targeting of SIK2/SIK3 holds the potential to restore immune balance by converting proinflammatory into regulatory pathways. Collectively, this work supports SIK2/SIK3 inhibition as a potential treatment strategy for myeloid cell–driven chronic inflammatory conditions.

## Abstract 2

Selective SIK2/SIK3 inhibition reprograms pathogenic myeloid cells from pro-inflammatory to immunoregulatory phenotypes, demonstrating their role in autoimmune diseases and offering a novel therapeutic approach.

## Introduction

Autoimmune diseases such as inflammatory bowel disease (IBD; including Crohn’s disease [CD] and ulcerative colitis [UC]), psoriasis (PsO), and rheumatoid arthritis (RA) are common chronic disorders that cause substantial disease burden worldwide (1). Uncontrolled chronic inflammation of the gastrointestinal tract, skin, or joints impairs patients’ well-being and physical function, and can lead to complications or irreversible structural damage of affected tissues. Although biologic and small-molecule-targeted therapies have transformed treatment, achievement of durable remission and long-term disease control remains a significant unmet clinical need (2, 3, 4, 5). These therapeutic limitations underscore the need for novel approaches that effectively control chronic inflammation.

Adaptive immune responses have traditionally been considered as the main drivers of chronic inflammation in autoimmune disease, but growing evidence indicates that dysregulated myeloid cells are key contributors to sustaining chronic inflammatory pathologies (6, 7). Mononuclear phagocytes, encompassing monocytes, macrophages, and dendritic cells (DCs), play pivotal roles in maintaining immune homeostasis by balancing immune activation and tolerance. Their plasticity allows them to adopt proinflammatory (M1-like) or antiinflammatory (M2-like) phenotypes in response to environmental cues, with timely transitions from inflammation to resolution being critical for tissue repair and homeostasis (6, 7). In contrast, this transition fails to appear in autoimmune and chronic inflammatory diseases, causing a chronic predominance of proinflammatory myeloid cells overpowering regulatory counterparts. The underlying mechanisms causing dysregulation are multifactorial, shaped by genetic predisposition, environmental triggers, immune abnormalities, and altered host-microbiota interactions (8). Tissue-resident macrophages and DCs in the skin and intestinal mucosa maintain immune homeostasis by adopting tolerogenic phenotypes that produce IL-10, a cytokine essential for suppressing excessive inflammation in response to commensal microorganisms, promoting the expansion of antigen-specific regulatory T cells (Tregs), and preserving tissue integrity. In the intestinal mucosa of IBD patients, impaired monocyte-macrophage differentiation leads to the accumulation of immature, proinflammatory macrophage and DC subsets with increased TLR expression, exaggerated microbial responses, and excessive secretion of proinflammatory cytokines such as TNF-α, IL-1β, IL-6, IL-12, and IL-23 (6, 7, 9). These cells perpetuate inflammation, recruit additional immune effectors, and drive tissue injury, while defective resolution further exacerbates disease progression (10). Myeloid DCs critically contribute to chronic inflammation by acting as key antigen-presenting cells that bridge innate and adaptive immunity. They capture antigens and pathogens and migrate to secondary lymphoid organs, where they activate T cells and further drive disease progression. Similar pathogenic roles are observed in PsO, where myeloid DCs amplify Th17-mediated responses via IL-23 signaling (11), and in RA, where the delicate M1/M2 imbalance contributes to synovitis and joint destruction (12, 13). Across these conditions, aberrant myeloid cell function is a consistent feature; however, it remains unclear whether this dysregulation is a cause or a consequence of persistent disease activity (7, 14).

Salt-inducible kinases (SIKs) are serine/threonine kinases with broad immunomodulatory functions across immune cell types. They belong to the AMP-activated protein kinase (AMPK) family and include 3 isoforms: SIK1, SIK2, and SIK3 (15, 16). SIK1 is inducible, upregulated by stimuli such as high salt intake and hormones, whereas SIK2 and SIK3 are constitutively expressed and more stably regulated. SIKs are widely expressed across tissues and regulate gene transcription by modulating nuclear translocation of histone deacetylases (HDACs) and cAMP response element–binding protein (CREB)–regulated transcription coactivators (CRTCs) (15–18).

In TLR- and IL-1R–activated myeloid cells, SIK inhibition through G protein–coupled receptor (GPCR) signaling or pharmacological inhibitors induces the nuclear translocation of HDACs and CRTCs. HDACs in the nucleus repress NF-κB–dependent expression of proinflammatory genes, while CRTCs enhance CREB-dependent transcription of antiinflammatory and immunoregulatory mediators (19). This cAMP/protein kinase A (PKA)–dependent regulation links GPCR signaling to tissue-specific gene expression in macrophages and DCs. The involvement of SIKs in modulating both pro- and antiinflammatory pathways was first demonstrated downstream of prostaglandin E2 (PGE2)–EP2/EP4 signaling in LPS-stimulated macrophages, and was later supported by studies showing that pan-SIK inhibition with nonselective kinase inhibitors mimicked these effects in activated macrophages and DCs (20–23). Additional studies using catalytically inactive SIK mutants and pharmacological SIK inhibitors have substantially advanced our understanding of the role of individual SIK isoforms, specifically highlighting SIK2 and SIK3 as dominant regulators of macrophage phenotypes (24). Independent studies have also identified a critical role for both SIK2 and SIK3 in thymic T cell development, although their function in peripheral T cell activation remains poorly understood (25). Further investigation into the roles of SIKs in both myeloid and T cells could provide valuable insights into their contributions to chronic inflammatory processes and help determine whether impaired myeloid cell regulation is a driver or a consequence of disease progression. Such insights could inform the development of targeted strategies to modulate pathogenic myeloid activity in autoimmune diseases.

Because SIK1 contributes minimally to myeloid cell polarization and is genetically associated with potential toxicities (26–30), therapeutic efforts are best directed toward SIK2 and SIK3 to enhance efficacy and minimize the risk of unwanted side effects. Through drug discovery and medicinal chemistry efforts, GLPG3970 was developed as a potent and selective first-in-class small-molecule inhibitor of SIK2 and SIK3 (IC_50_ of 282.8 nM for SIK1, 7.8 nM for SIK2, and 3.8 nM for SIK3 in biochemical assays) (31). GLPG3970 demonstrates high selectivity across the human kinome and has been optimized with a favorable preclinical safety and pharmacological profile (31). In a phase I study (NCT04106297) conducted in healthy participants, once-daily doses of GLPG3970 up to 600 mg were well tolerated and demonstrated a favorable pharmacokinetic profile (32), supporting its advancement into patient studies. Robust target engagement was observed at 400 mg and 600 mg, as evidenced by inhibition of plasma TNF-α and induction of IL-10 in an ex vivo LPS-stimulated human whole blood assay (31).

Our research focused on the immunomodulatory effects of selectively inhibiting SIK2 and SIK3 across key immune cell types and in models of immune-driven pathology. Using disease-relevant in vitro systems, we evaluated the SIK2/SIK3 inhibitor GLPG3970 in myeloid, T, and B cells and examined its effects in preclinical models of inflammation to define its therapeutic potential and elucidate mechanisms of action at molecular and cellular levels within affected tissues. These preclinical findings were subsequently translated into 3 parallel, placebo-controlled, 6-week signal-detection studies in patients with moderate to severe UC, PsO, and RA, designed to assess early biological activity, safety, and tolerability. Collectively, this work provides a foundation for understanding how SIK2/SIK3 inhibition modulates the persistent activation and function of inflammatory macrophages and DCs and highlights SIK2/SIK3 inhibitors as a strategy to reprogram myeloid cells from proinflammatory toward immunoregulatory states, supporting their potential as treatments for chronic inflammatory diseases such as UC and PsO.

## Results

### SIK2/SIK3 inhibition reprograms activated monocytes and macrophages

To examine whether SIK2 and SIK3 play a pivotal role in regulating monocyte and macrophage differentiation toward pro- or antiinflammatory phenotypes, we investigated the effects of GLPG3970 on activated monocytes and monocyte-derived macrophages in vitro.

**Figure 1:**
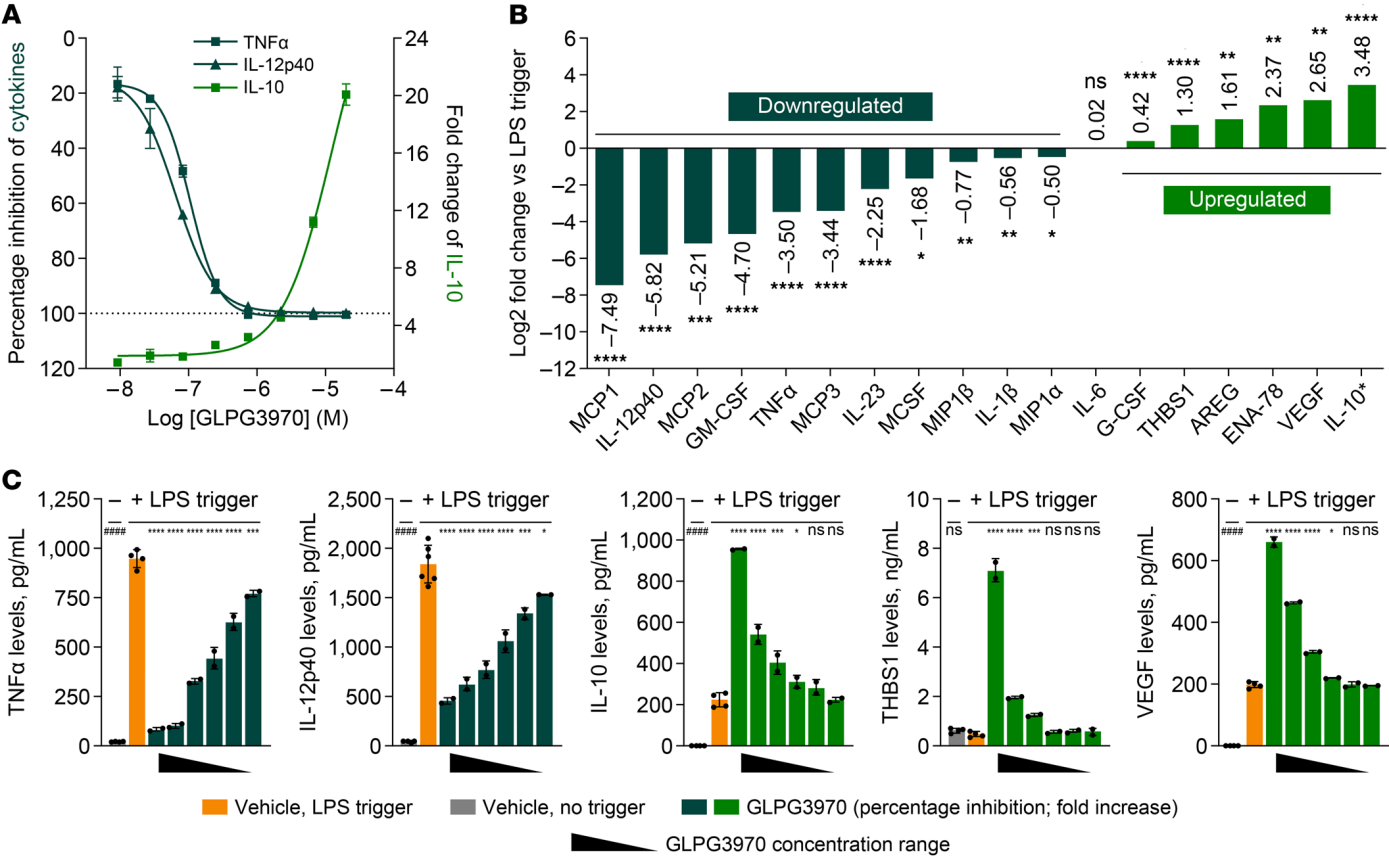
GLPG3970 reprograms proinflammatory monocytes and macrophages. (**A**) Inhibition of TNF-α (4 hours) and IL-12p40 (20 hours) and induction of IL-10 (4 hours) by GLPG3970 in LPS-stimulated human monocytes (31). Data shown are mean percentage inhibition of TNF-α and IL-12p40 and mean fold change of IL-10 relative to LPS. (**B**) Effects of GLPG3970 on a panel of cytokines and chemokines in LPS-stimulated monocytes. Data are log2 fold change versus LPS. All analytes were measured at 24 hours, except for IL-10* (4 hours). GLPG3970 was tested at 5 or 6.67 μM. (**C**) GLPG3970 inhibition of TNF-α and IL-12p40 and induction of IL-10, THBS1, and VEGF in LPS-stimulated monocyte-derived macrophages. Data are from 2 donors, each with 2 biological replicates. Cytokines were measured at 24 hours, except for IL-10 (4 hours). Macrophages were treated with 20, 6.7, 2.2, 0.74, 0.25, or 0.08 μM GLPG3970. Statistical analyses versus LPS controls were performed using paired *t* tests or 1-way ANOVA with Dunnett’s correction on non-log-transformed data. ^####^*P* < 0.0001; ^ns^*P* > 0.05; *P < 0.05, **P < 0.01, ***P < 0.001, ****P < 0.0001. AREG, amphiregulin; ENA-78, epithelial-derived neutrophil-activating peptide 78; G-CSF, granulocyte colony-stimulating factor; M-CSF, macrophage colony-stimulating factor; MCP, monocyte chemoattractant protein; MIP, macrophage inflammatory protein; THBS1, thrombospondin 1.

Human primary monocytes and monocyte-derived macrophages were stimulated with LPS in the presence or absence of GLPG3970 to model disease-relevant activation conditions. In LPS-stimulated monocytes, GLPG3970 potently inhibited TNF-α and IL-12p40 production, with mean half-maximal inhibitory concentration (IC_50_) values of 231 nM (TNF-α; negative log IC_50_ [pIC_50_] = 6.64 ± 0.10, *n* = 5 donors) and 67 nM (IL-12p40; pIC_50_ = 7.17 ± 0.09, *n* = 5 donors) (Figure 1A) (31). GLPG3970 also increased IL-10 secretion, with early onset and peak induction observed at 4 hours of LPS stimulation (fold change at 20 μM = 13.8 ± 1.9, *n* = 4; significant induction at 1,700 nM compared with LPS controls, 1-way ANOVA; Figure 1A).

Analysis of a broader cytokine and chemokine panel showed that GLPG3970 robustly inhibited multiple proinflammatory mediators, including MCP-1, MCP-2, MCP-3, GM-CSF, M-CSF, MIP-1α, MIP-1β, TNF-α, IL-12p40, IL-23, and IL-1β, with no effect on IL-6 levels (Figure 1B). In parallel, GLPG3970 enhanced the production of several antiinflammatory and pro-resolving mediators, including THBS1, G-CSF, AREG, ENA-78, VEGF, and IL-10 (Figure 1B).

Inhibition of SIK2 and SIK3 with GLPG3970 in LPS-stimulated monocyte-derived macrophages increased the secretion of IL-10 while strongly reducing the production of proinflammatory cytokines, including TNF-α and IL-12p40 (Figure 1C). In addition, GLPG3970 induced the release of pro-resolving mediators such as THBS1, VEGF, and AREG in a dose-dependent manner (data on AREG not shown).

### SIK2/SIK3 inhibition induces a tolerogenic DC phenotype

**Figure 2:**
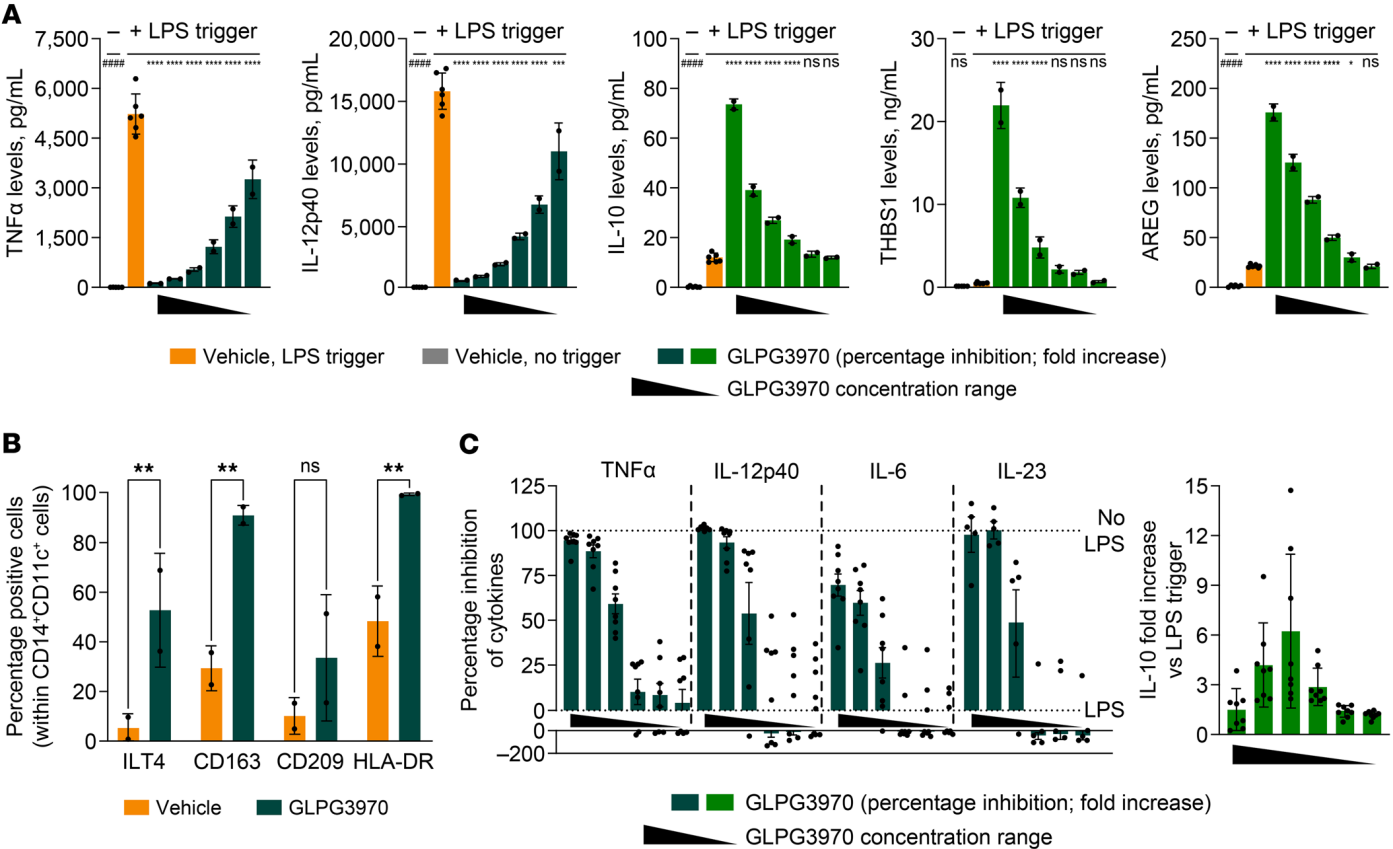
GLPG3970 induces tolerogenic DCs. (**A**) Inhibition of TNF-α and IL-12p40 and induction of IL-10, THBS1, and AREG by GLPG3970 in LPS-stimulated human monocyte-derived DCs. After differentiation, DCs were treated with GLPG3970 at 20, 6.7, 2.2, 0.74, 0.25, or 0.08 μM. Cytokines were measured at 24 hours after LPS stimulation, except for IL-10 (4 hours). Data were collected from 2 independent donors, each with 2 biological replicates. (**B**) Expression of DC surface markers (ILT4, CD163, CD209, HLA-DR) on CD14^+^CD11c^+^ cells after 5 days of differentiation in the presence of 2.2 μM GLPG3970. Data are presented as mean values from 2 donors. (**C**) Percentage inhibition of TNF-α, IL-12p40, IL-6, and IL-23 and fold induction of IL-10 in LPS-stimulated DCs differentiated in the presence of GLPG3970 (2.2, 0.7, 0.2, 0.08, 0.03, 0.001 μM). Cytokines were measured 4 hours after LPS stimulation. Sample sizes: *n* = 8 donors for TNF-α, IL-12p40, IL-6, and IL-10; *n* = 5 for IL-23. Statistical comparisons: (**B**) GLPG3970 vs. vehicle by paired *t* test with FDR correction; (**A** and **C**) 1-way ANOVA with Dunnett’s multiple comparisons vs. LPS controls. ^####^*P* < 0.0001; ^ns^*P* > 0.05; *P < 0.05, **P < 0.01, ***P < 0.001, ****P < 0.0001. AREG, amphiregulin; HLA-DR, human leukocyte antigen–DR isotype; ILT4, immunoglobulin-like transcript 4; THBS1, thrombospondin 1.

Given their central role in regulating immune responses, we next investigated the effects of GLPG3970 on monocyte-derived DCs (33). Short-term exposure of fully differentiated DCs to GLPG3970 followed by LPS stimulation resulted in dose-dependent suppression of TNF-α and IL-12p40 production and enhanced release of IL-10, THBS1, and AREG in comparison with vehicle controls (Figure 2A).

To assess the impact of GLPG3970 during DC differentiation, monocytes were cultured in the presence of GLPG3970 for 5 days before phenotypic characterization by flow cytometry. GLPG3970 increased the proportion of CD14^+^CD11c^+^ cells expressing markers associated with a tolerogenic phenotype, including ILT4 and CD209, as well as markers linked to regulatory or immunosuppressive functions, such as CD163 and HLA-DR (Figure 2B) (34–36).

Functional analysis of DCs differentiated in the presence of GLPG3970 showed a stable tolerogenic profile, characterized by markedly reduced production of TNF-α, IL-6, IL-12p40, and IL-23, coupled with potent induction of IL-10 release upon LPS stimulation after GLPG3970 washout (Figure 2C). GLPG3970 induced a stable tolerogenic profile at lower concentrations when administered during cell differentiation, compared with GLPG3970 addition after differentiation, indicating phenotypic reprogramming rather than merely inhibition of LPS stimulation.

### SIK2/SIK3 inhibition dampens T cell activation and induces an IL-10–producing regulatory B cell phenotype

**Figure 3:**
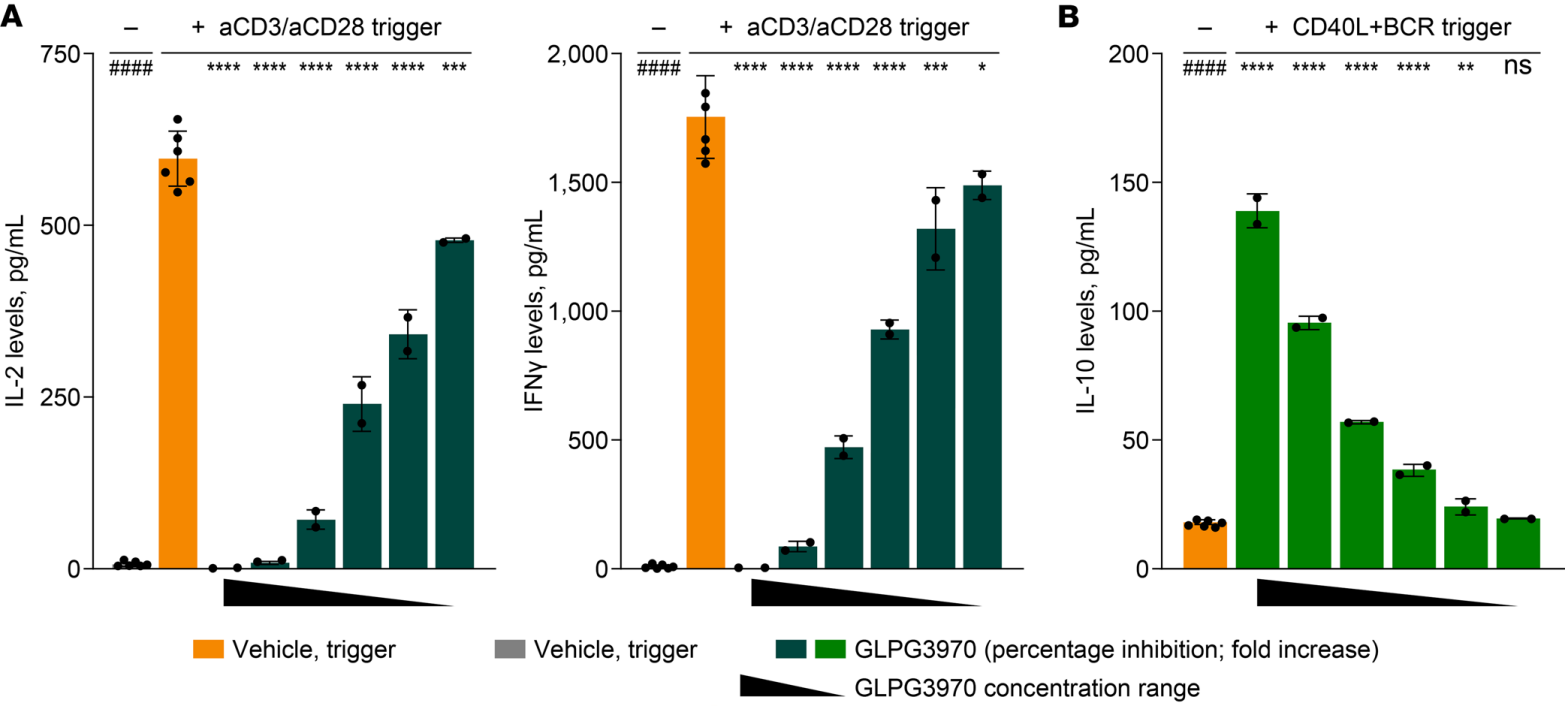
SIK2/SIK3 inhibition dampens T cell activation and induces IL-10–producing B cells. (**A**) Inhibition of IL-2 and IFN-γ production by GLPG3970 in immune-activated T cells. Human PBMCs were stimulated with anti-CD3/anti-CD28 for 24 hours in the presence of GLPG3970 (20, 6.7, 2.2, 0.74, 0.25, or 0.08 μM). Data were collected from 4 independent donors, each with 2 biological replicates. (**B**) Induction of IL-10 by GLPG3970 in human tonsillar B cells. Cells were stimulated with CD40L/anti-BCR for 3 days in the presence of GLPG3970 (20, 6.7, 2.2, 0.74, 0.25, or 0.08 μM). Data were collected from 4 independent donors, each with 2 biological replicates. Statistical analyses versus triggered controls were performed using 1-way ANOVA with Dunnett’s multiple-comparison test. ^####^*P* < 0.0001; ^ns^*P* > 0.05; *P < 0.05, **P < 0.01, ***P < 0.001, ****P < 0.0001. BCR, B cell receptor.

To date, the effect of SIK2/SIK3 inhibition on T and B cell activation remains largely unexplored. Treatment of human peripheral blood mononuclear cells (PBMCs) with GLPG3970 following activation using anti-CD3/anti-CD28 antibodies resulted in dose-dependent inhibition of T cell cytokines, including IL-2 and interferon-γ (IFN-γ) (Figure 3A). Complete suppression of IL-2 and IFN-γ required higher concentrations of GLPG3970 (20 μM) compared with the concentrations sufficient to inhibit proinflammatory cytokine production in myeloid cells (6.7 μM).

IL-10–producing regulatory B cells (Bregs) are important modulators of immune responses (37). Stimulation of human primary tonsillar B cells with CpG-B, a TLR9 agonist, induces IL-10–producing Bregs (38), and CREB has been identified as a key transcription factor driving their differentiation (39). We first investigated whether GLPG3970 could induce IL-10 production in B cells, given that SIK2/SIK3 inhibition results in the activation of CREB-dependent gene transcription. Human primary tonsillar B cells were activated in a 3-day assay using a combination of CD40L and anti-IgG/IgM to simultaneously engage CD40 and the B cell receptor (BCR). Treatment with GLPG3970 resulted in a dose-dependent increase in IL-10 secretion (Figure 3B). RNA sequencing of CD40L/anti-BCR–stimulated tonsillar B cells confirmed that CpG-B treatment induced a Breg phenotype, characterized by enrichment of gene sets previously associated with mouse Bregs (Supplemental Figure 1A; supplemental material available online with this article; https://doi.org/10.1172/jci.insight.171776DS1) (40). Moreover, GLPG3970 treatment of CD40L/anti-BCR–activated B cells similarly elicited a Breg-like transcriptomic signature that correlated with the CpG-B–induced Breg gene expression profile (Supplemental Figure 1, B and C).

### SIK2/SIK3 inhibition attenuates disease activity in mouse colitis models

To evaluate whether SIK2/SIK3 inhibition can reprogram proinflammatory myeloid cells in vivo, we investigated the effects of GLPG3970 in preclinical mouse models of experimental colitis (41, 42). The translational relevance of SIK inhibition from human to murine systems is supported by the high conservation of SIK kinase domains across vertebrate species (16).

Mechanistic validation of GLPG3970 was first established in an acute LPS-induced cytokine release model, where treatment resulted in dose-dependent inhibition of plasma TNF-α and a concomitant increase in plasma IL-10, consistent with in vitro findings in LPS-stimulated myeloid cells. In an acute dextran sulfate sodium–induced colitis model, prophylactic administration of GLPG3970 significantly reduced disease activity scores and improved histopathological outcomes (31).

To ensure sustained target engagement in vivo, GLPG3970 was administered twice daily via oral gavage, accounting for its short plasma half-life of approximately 0.55 hours in mice. Earlier research with GLPG3970 showed that twice-daily dosing at 30 mg/kg maintained plasma concentrations above the TNF-α IC_50_ threshold for up to 16 hours, as established in the acute LPS challenge model, thereby supporting the twice-daily dosing regimen used in preclinical mouse studies in this study (31).

**Figure 4:**
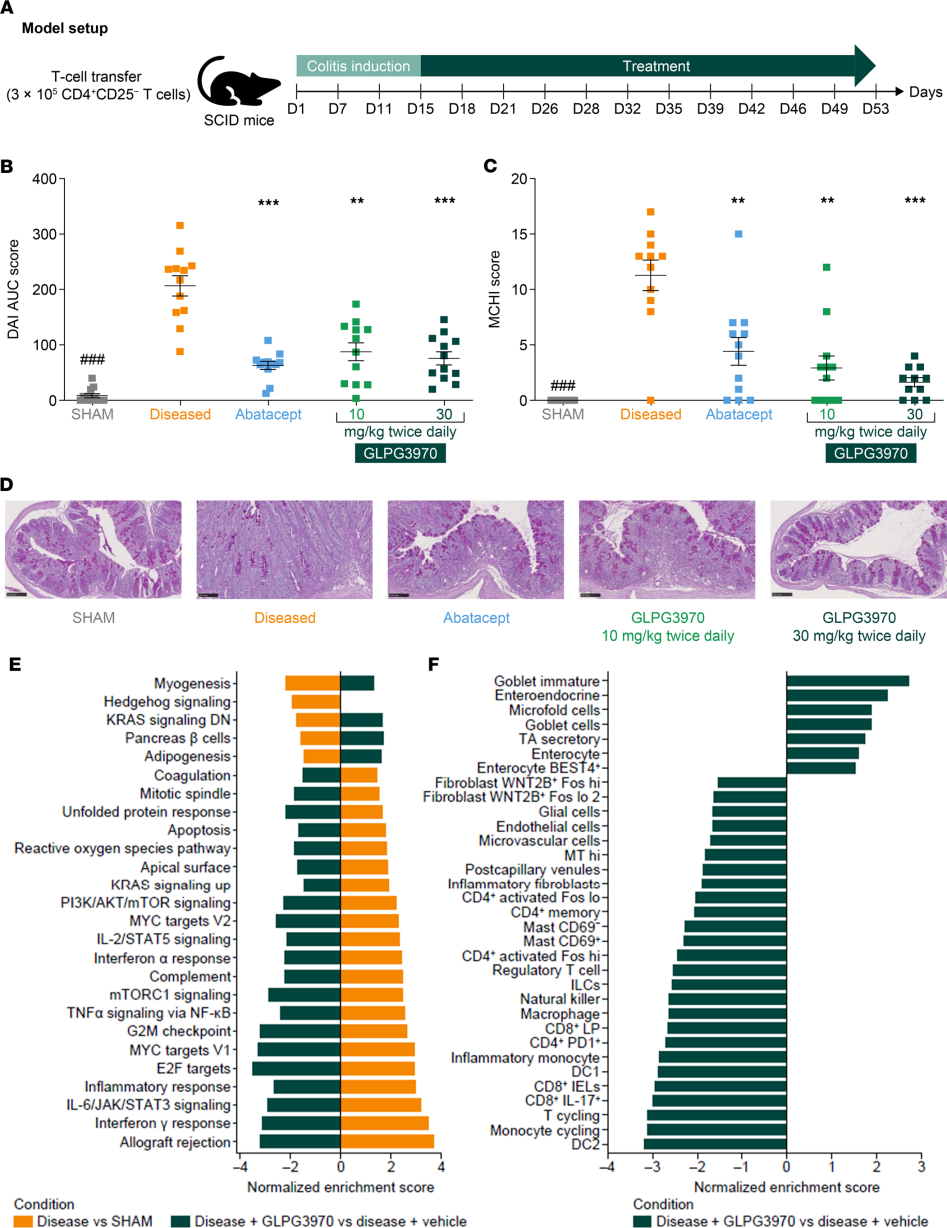
SIK2/SIK3 inhibition attenuates disease in T cell transfer colitis model. (**A**) Experimental design of T cell transfer colitis model. SCID (CB17) mice received 3 × 10^5^ CD4^+^CD25^–^ T cells isolated from BALB/c mice on day 1 to induce colitis. From day 15 to day 53, T cell–grafted animals were treated orally with GLPG3970 (10 or 30 mg/kg, twice daily). Abatacept (10 mg/kg, i.p., 3 times weekly) was included as an active control. (**B** and **C**) AUC of disease activity index (DAI) score (recorded every 3–4 days; a composite score of body weight loss, stool consistency, and rectal bleeding) (**B**) and Mouse Colitis Histology Index (MCHI) score (**C**). Data are presented as mean ± SEM of AUC DAI and MCHI scores (*n* = 12 mice per group). (**D**) Representative periodic acid–Schiff–stained (PAS-stained) colon sections. Scale bars: 100 μm. (**E**) Gene set enrichment analysis of colon samples. Shown are hallmark pathways significantly upregulated (normalized enrichment score [NES] > 0) or downregulated (NES < 0) in diseased versus healthy animals (orange) and in GLPG3970-treated versus diseased vehicle animals (green). Bar plots display significantly modified pathways (adjusted *P* < 0.05) ranked by NES. (**F**) Cell type enrichment analysis. Enrichment of colon cell type marker signatures, as described in ref. 61, among genes differentially expressed with GLPG3970 treatment (GLPG3970 vs. diseased vehicle). Significance cutoff: adjusted *P* < 0.05. For **C**, statistical significance versus diseased vehicle was assessed by 1-way ANOVA with Dunnett’s multiple-comparison test (MCHI data log-transformed, control group excluded). For **E** and **F**, adjusted *P* < 0.05 was used to define significant enrichment. ^###^*P* < 0.001; *P < 0.05, **P < 0.01, ***P < 0.001.

Here, we investigated GLPG3970 effects in 2 more stringent chronic mouse colitis models. In the adoptive T cell transfer model, colitis is induced by transfer of a Treg-depleted T cell fraction into mice with severe combined immunodeficiency (SCID). Efficacy was evaluated in a therapeutic setting, with GLPG3970 treatment initiated once disease activity was established and continued for 5 weeks (Figure 4A) (41). Oral administration of GLPG3970 (10 or 30 mg/kg, twice daily) significantly reduced disease activity index (DAI) (AUC day 15–53; 10 mg/kg *P* < 0.01, 30 mg/kg *P* < 0.001; Figure 4B) compared with abatacept used as positive control (CTLA4-Ig, a well-described efficacious agent in this model) (43). DAI is a composite score encompassing body weight loss, stool consistency, and fecal blood. Histological assessment using the Mouse Colitis Histology Index (MCHI) revealed significant improvements in colonic tissue integrity (MCHI: 10 mg/kg *P* < 0.01, 30 mg/kg *P* < 0.001; Figure 4C). The MCHI is a composite histological score that integrates multiple subscores reflecting epithelial integrity, mucosal architecture, and inflammatory burden, and closely correlates with histopathological indices used in clinical studies of patients with IBD (44). These findings were corroborated by periodic acid–Schiff (PAS)–stained sections showing preservation of goblet cells (mucin-producing areas), reduced inflammatory infiltrates, and decreased crypt loss (Figure 4D).

In a second colitis model, genetic Mdr1a^−/−^ mice spontaneously develop colitis, which is further exacerbated by subsequent infection with *Helicobacter bilis* (Supplemental Figure 2A) (45). In this model, oral treatment with GLPG3970 (10 or 30 mg/kg, twice daily) similarly reduced progressive colitis as recorded by DAI scores (AUC day 1–38; 10 and 30 mg/kg *P* < 0.05; Supplemental Figure 2B) and improved MCHI scores (10 and 30 mg/kg *P* < 0.05; Supplemental Figure 2C) compared with the anti–IL-12p40 antibody positive control. PAS-stained sections confirmed protection from colonic tissue damage and disease progression (Supplemental Figure 2D).

### GLPG3970 modulates myeloid cell and cytokine profiles within inflamed colon tissues

To investigate the mechanisms underlying GLPG3970 efficacy, we analyzed molecular and cellular changes in colonic tissues from the adoptive T cell transfer colitis model. Transcriptomic analysis identified 1,240 upregulated and 1,146 downregulated genes in diseased versus healthy colons (log2 fold change > 1, adjusted *P* < 0.05; Supplemental Figure 3A). Oral administration of GLPG3970 (30 mg/kg twice daily) reversed most disease-associated transcriptional changes, modulating 1,320 genes (549 up- and 771 downregulated; log2 fold change > 1, adjusted *P* < 0.05; Supplemental Figure 3B) and showing a Spearman’s correlation of –0.83 relative to disease-induced expression. Functional enrichment analysis revealed activation of multiple inflammatory gene sets during disease, including type I/II interferon, IL-6/JAK-STAT, and TNF-α/NF-κB signaling pathways, of which 25 of 26 were counter-regulated by GLPG3970 (Figure 4E). Cell type marker enrichment analysis showed downregulation of markers for inflammatory monocytes and type 2 DCs, along with upregulation of epithelial cell markers, including enterocytes and goblet cells (Figure 4F and Supplemental Figure 3C).

**Figure 5:**
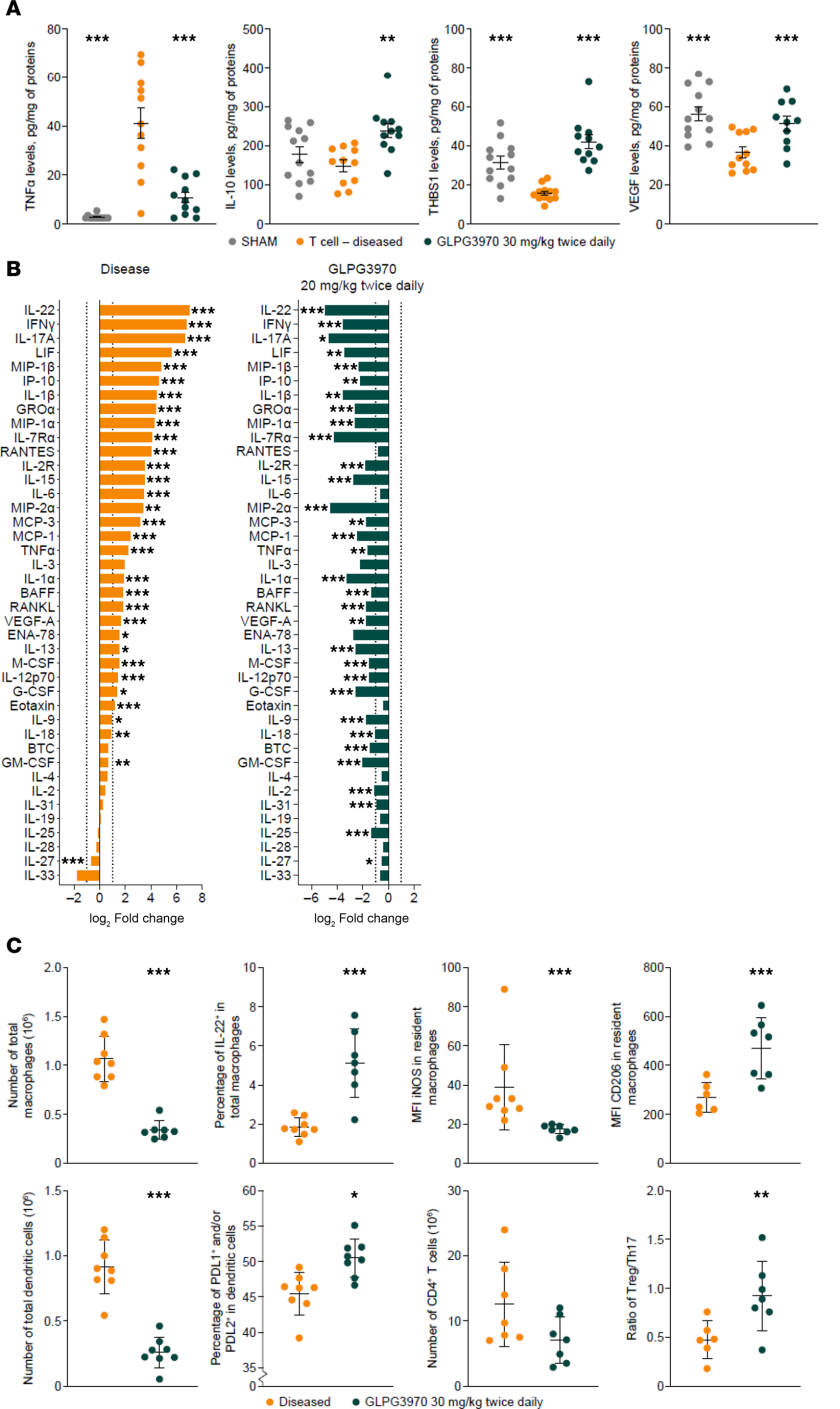
SIK2/SIK3 inhibition attenuates disease in T cell transfer colitis model. (**A**) Individual quantification of cytokines and pro-resolving mediators. Levels of TNF-α, IL-10, THBS1, and VEGF were measured by ELISA in colon tissue lysates from sham, diseased vehicle, and GLPG3970-treated (30 mg/kg twice daily) animals. Data expressed as pg/mg of total protein. (**B**) Broad panel of cytokine and chemokine profiling. A panel was measured in colon lysates from an independent T cell transfer study with GLPG3970 (20 mg/kg twice daily). Data expressed as log2 fold change versus sham (disease effect) and versus diseased vehicle (GLPG3970 treatment effect). (**C**) Immunophenotyping of lamina propria mononuclear cells from colon tissues. Top: Total macrophages per colon, frequency of IL-22^+^ macrophages, and expression levels (MFI) of inducible nitric oxide synthase (iNOS) and CD206 in resident macrophages. Bottom: Total DCs and CD4^+^ T cells, frequency of programmed death ligand 1–positive (PD-L1^+^) and/or PD-L2^+^ tolerogenic DCs, and ratio of Tregs to Th17 cells. For **A**–**C**, statistical analyses were performed using 1-way ANOVA with Dunnett’s multiple-comparison test vs. diseased vehicle. ^###^*P* < 0.001; *P < 0.05, **P < 0.01, ***P < 0.001.

Protein analysis of colonic lysates collected at study termination confirmed significant reductions in TNF-α (*P* < 0.001) and increases in IL-10 (*P* < 0.01), THBS1 (*P* < 0.001), and VEGF (*P* < 0.001) in GLPG3970-treated mice (30 mg/kg twice daily; Figure 5A). Multiplex cytokine/chemokine profiling revealed broad decreases in proinflammatory mediators in comparison with vehicle controls (Figure 5B).

Flow cytometric analysis of lamina propria mononuclear cells isolated from colonic tissues showed that GLPG3970 decreased total macrophage numbers. Intestinal macrophages were identified by coexpression of F4/80 and CD64, with resident macrophages further distinguished by high CX3CR1 expression (46). GLPG3970 shifted resident macrophages from an M1 (iNOS^+^) to an M2 (CD206^+^) profile and increased the percentage of IL-22^+^ macrophages (9, 47). Intestinal DCs were also analyzed, including tolerogenic CD3^–^CD11c^+^ DCs expressing PD-L1 and/or PD-L2 (48). GLPG3970 reduced the total number of DCs and promoted a tolerogenic phenotype. Total CD4^+^ T cell numbers remained unchanged, whereas the Treg-to-Th17 ratio increased, primarily owing to a reduction in Th17 cell percentages (Figure 5C). Detailed gating strategies used for the flow cytometric analyses are provided in Supplemental Figure 4, A–F.

### Activity of GLPG3970 in preclinical disease models of PsO and arthritis

**Figure 6:**
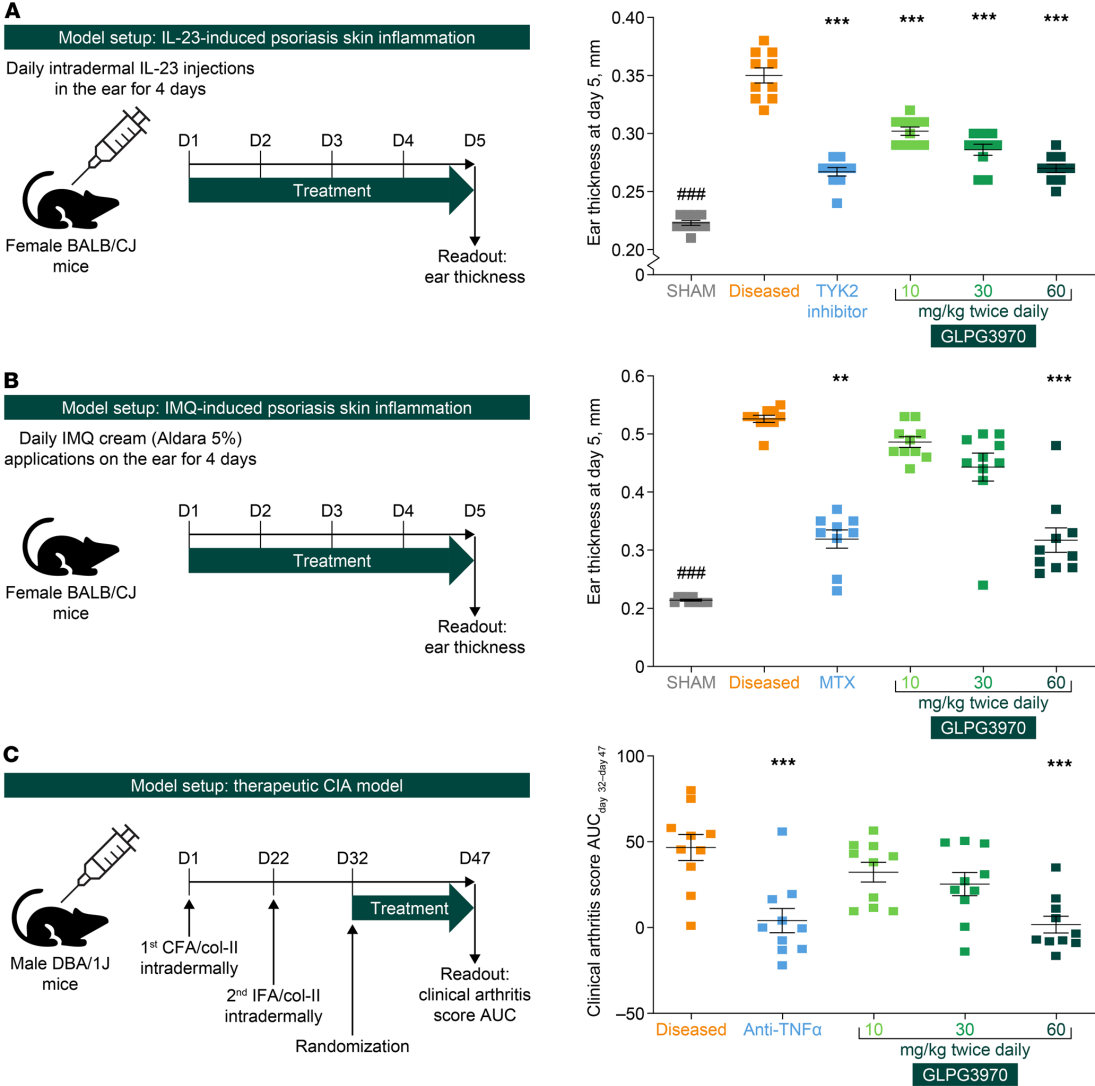
GLPG3970 attenuates PsO-like skin inflammation and arthritis in mouse disease models. (**A**) Ear thickness in the IL-23–induced PsO-like skin inflammation model measured on day 5. (**B**) Ear thickness in the imiquimod (IMQ)-induced PsO-like skin inflammation model measured on day 5. (**C**) Clinical arthritis score (AUC) from day 32 to day 47 in the therapeutic collagen-induced arthritis (CIA) model. Data are presented as mean ± SEM (*n* = 10 mice per group). Statistical analyses were performed using 1-way ANOVA with multiple-comparison test versus disease vehicle. ^###^*P* < 0.001; **P < 0.01, ***P < 0.001. IFA, incomplete Freund’s adjuvant; MTX, methotrexate; TYK2, tyrosine kinase 2.

The broader therapeutic potential of SIK2/SIK3 inhibition was evaluated in preclinical models of PsO-like skin inflammation and collagen-induced arthritis. In the mouse IL-23–induced PsO model, GLPG3970 significantly reduced ear thickening, measured on day 5, at all tested doses (10, 30, and 60 mg/kg twice daily, all *P* < 0.001; Figure 6A), demonstrating a dose-dependent antiinflammatory effect comparable to that of a TYK2 inhibitor (*P* < 0.001) (49). In the imiquimod (IMQ)-induced model, GLPG3970 significantly reduced ear thickness at 60 mg/kg (*P* < 0.001; Figure 6B), to an extent similar to that of methotrexate (*P* < 0.01). Neutrophil infiltration in skin tissue sections was also diminished by GLPG3970 at 30 and 60 mg/kg (both *P* < 0.001; Supplemental Figure 5, A and B). Overall, SIK2/SIK3 inhibition effectively suppressed skin inflammation in both PsO models, mirroring the concentration-to-effect relationship observed in the colitis models.

In the collagen-induced arthritis (CIA) model, therapeutic administration of GLPG3970 (10, 30, and 60 mg/kg twice daily) after disease onset reduced clinical scores in a dose-dependent manner, with significant effects observed only at the highest dose (60 mg/kg twice daily, *P* < 0.001 vs. diseased group; Figure 6C). The effect size at 60 mg/kg was comparable to that of the anti–TNF-α antibody positive control (*P* < 0.001; Supplemental Figure 6A). Radiographic assessment revealed that GLPG3970 at 60 mg/kg significantly prevented bone erosion, as measured by Larsen scores, relative to the positive control (*P* < 0.05; Supplemental Figure 6B). GLPG3970 exposure levels at 60 mg/kg twice daily, which corresponded to anti–TNF-α efficacy in the CIA model, exceeded the exposure levels achieved with 10 and 30 mg/kg twice daily doses that were effective in PsO and colitis models, indicating that higher exposure levels may be required in RA compared with UC and PsO.

### GLPG3970 in clinical studies

#### Clinical signal-detection studies in UC, PsO, and RA.

GLPG3970 was evaluated at 350–400 mg once daily in 3 exploratory, 6-week signal-detection studies in patients with moderately to severely active UC, PsO, and RA. Dose selection was informed by preclinical efficacy and target engagement observed in an ex vivo LPS challenge assay during first-in-human studies. In phase I studies, GLPG3970 demonstrated favorable pharmacokinetics with an elimination half-life of 9.5–15.0 hours, acceptable safety, and robust target engagement, supporting once-daily dosing (32). Although GLPG3970 doses up to 600 mg were tested in healthy volunteers, the clinical dose was limited to 400 mg to limit off-target inhibition of the hERG potassium channel (IC_50_ = 15.3 μM) (31), thus mitigating QTc prolongation and risk of potentially lethal arrhythmia. While no serious adverse events were reported, the dose cap reflects a safety consideration intrinsic to GLPG3970’s pharmacology. As these were small, early-phase, hypothesis-generating trials, none were powered to formally assess clinical efficacy.

**Figure 7:**
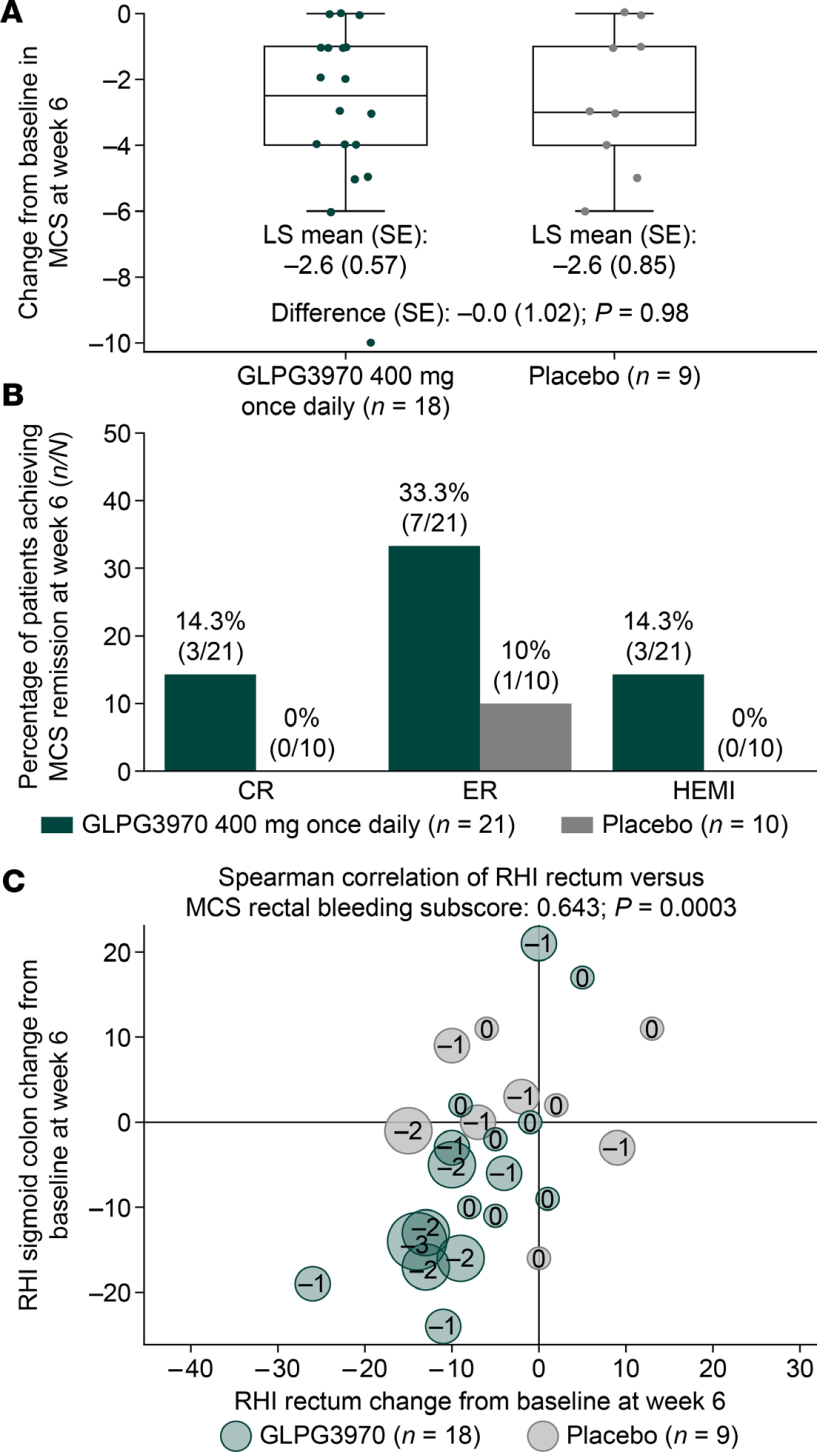
Clinical outcomes with GLPG3970 in patients with UC. (**A**) Least-squares (LS) mean change from baseline at week 6 in Mayo Clinic Score (MCS) in patients with data available post-baseline. (**B**) Percentage of patients achieving clinical remission (CR; defined as a decrease of ≥3 points and ≥30% from baseline in MCS, and a decrease in rectal bleeding subscore of ≥1 or an absolute rectal bleeding subscore of 0 or 1), endoscopic response (ER; Mayo endoscopic subscore 0 or 1), and improvement in histologic-endoscopic mucosal index (HEMI; Mayo endoscopic subscore 0 or 1 and Robarts Histological Index [RHI] ≤ 3, where RHI is the average of rectum and sigmoid colon RHIs) at week 6. Nonresponder imputation was used for patients with missing data. (**C**) Change from baseline in RHI of rectum versus sigmoid colon at week 6. Bubble size corresponds to improvement from baseline in MCS–rectal bleeding subscore. Spearman’s correlation between RHI rectum score and change in MCS–rectal bleeding subscore is shown. Statistical analyses: LS mean changes and proportions were calculated for patients with available data. Spearman’s correlation was used to assess association between RHI rectum score and MCS–rectal bleeding improvement.

For UC, 31 patients with moderate/severe disease and inadequate response to conventional therapies were randomized to once-daily GLPG3970 400 mg (*n* = 21) or placebo (*n* = 10; NCT04577794), with 29 patients completing treatment (Supplemental Figure 7). Baseline mean Mayo Clinic Score (MCS) was 8.5 versus 8.2 in the GLPG3970 and placebo groups, respectively (Supplemental Table 1). However, patients randomized to active treatment had more severe activity than the placebo group, indicated by higher MCS–endoscopic subscore, histological activity, and fecal calprotectin values compared with the placebo group (Supplemental Table 1). After 6 weeks, the primary endpoint was not met (least-squares [LS] mean MCS change –2.6 [standard error (SE) 0.57] for GLPG3970 vs. –2.6 [SE 0.85] for placebo; difference 0.0 [SE 1.02]; *P* = 0.981; Figure 7A). However, consistent improvements were observed in rectal bleeding, endoscopic subscore, MCS remission, and histologic-endoscopic mucosal index (HEMI) (Figure 7B). A post hoc analysis showed a significant correlation between Robarts Histological Index reduction and rectal bleeding improvement (Spearman’s correlation value of 0.643, *P* = 0.0003; Figure 7C). Safety outcomes are described in Supplemental Results and summarized in Supplemental Table 2.

**Figure 8:**
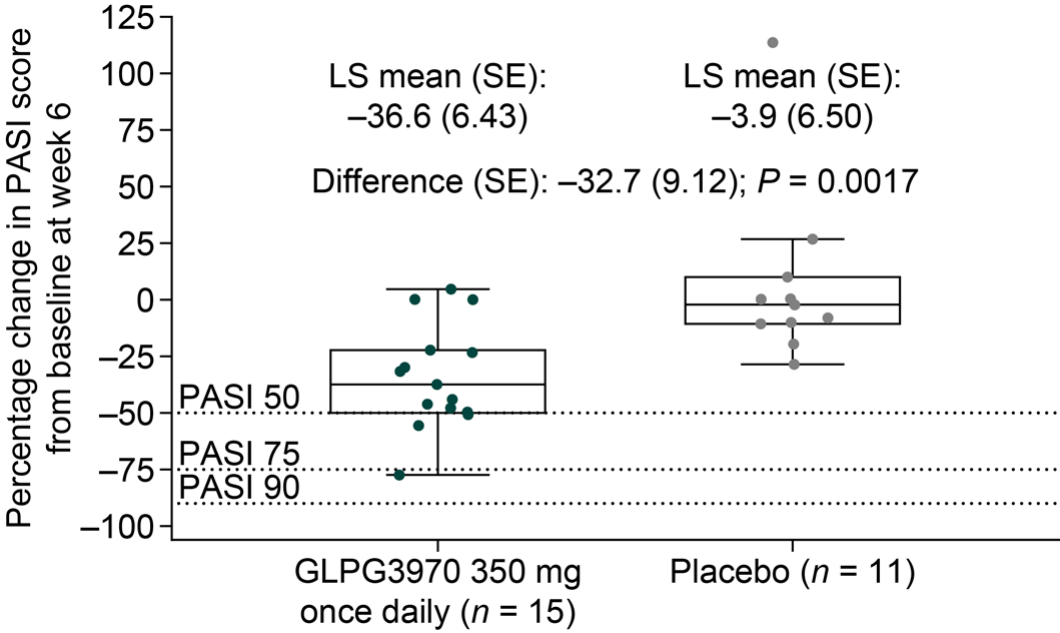
Clinical outcomes with GLPG3970 in patients with PsO. (**A**) Least-squares (LS) mean change from baseline in Psoriasis Area and Severity Index (PASI) score at week 6 (or last observation post-baseline in the case of early study discontinuation, *n* = 3 patients). PASI50, PASI75, and PASI90 responses are defined as ≥50%, ≥75%, and ≥90% reduction in PASI score, respectively, relative to baseline. Statistical analyses: LS mean changes and response rates were calculated for patients with available data using nonresponder imputation for missing values.

For PsO, 26 patients with moderate to severe plaque PsO were randomized to once-daily 350 mg GLPG3970 (*n* = 15) or placebo (*n* = 11; NCT04106297), with 23 patients completing treatment (Supplemental Figure 8). Baseline median Psoriasis Area and Severity Index (PASI) scores were 15.6 and 16.1, respectively (Supplemental Table 3). At week 6, the LS mean (SE) percentage change in PASI score from baseline was significantly decreased by –36.6% (6.43) in the GLPG3970 group versus –3.9% (6.50) in the placebo group, with a difference of –32.7% (9.12; *P* = 0.0017; Figure 8). Three patients achieved PASI50, including one PASI75 responder. Safety outcomes are described in Supplemental Results and summarized in Supplemental Table 2.

For RA, 28 patients were treated with GLPG3970 400 mg once daily (*n* = 16) or placebo (*n* = 12; NCT04577781), with 23 patients completing treatment (Supplemental Figure 9). At baseline, mean disease activity score based on 28 joint counts and C-reactive protein (DAS28-CRP) values were 6.13 versus 5.68, respectively (Supplemental Table 4). The primary endpoint was not met; DAS28-CRP values slightly decreased in both study groups, reaching an LS mean (SE) percentage change from baseline of –1.3% (0.22) for GLPG3970 versus –1.2% (0.26) for placebo (difference −0.1% [0.35]; *P* = 0.89; Supplemental Figure 10). Secondary endpoints, including C-reactive protein (CRP) levels and American College of Rheumatology (ACR) 20/50/70 responses (the latter representing 20%, 50%, or 70% improvement from baseline in the ACR score, respectively), confirmed the lack of clinical efficacy at this dose. Baseline demographics and safety outcomes are described in Supplemental Table 4 and Supplemental Table 2, respectively.

#### GLPG3970 exposure.

Population pharmacokinetics modeling, based on data from healthy participants, predicted average free plasma concentrations (C_ave_) of 235 nM and 275 nM for once-daily doses of 350 mg and 400 mg GLPG3970, respectively. Sparse pharmacokinetic data from 3 patient studies showed C_trough_ levels consistent with those in healthy participants (Galapagos, data on file). However, free C_ave_ in patients with PsO, UC, and RA was 1.8- to 11.3-fold lower than the exposures associated with minimal effective doses in the corresponding mouse models (PsO, colitis, and CIA) (Table 1).

#### Overall safety data.

No serious adverse events or deaths were reported in any of the studies. All GLPG3970 treatment-emergent adverse events were mild or moderate in severity. Clinically significant laboratory parameters reported in more than 1 patient treated with GLPG3970 were increased amylase and lipase. Further details on safety data are available in Supplemental Results and Supplemental Table 2.

## Discussion

Our findings provide evidence supporting selective SIK2/SIK3 inhibition as a potential immunomodulatory strategy for chronic inflammatory diseases. GLPG3970 promoted reprogramming of human myeloid cells both in vitro and in vivo in mice toward an antiinflammatory phenotype and function, aligning with its therapeutic efficacy across multiple preclinical models of immune-mediated disease. Moreover, in exploratory clinical studies, GLPG3970 treatment demonstrated signals of efficacy in patients with UC and PsO, despite achieving sub-therapeutic systemic exposures. Together, these findings support the broader therapeutic potential of SIK2/SIK3 inhibition and provide a rationale for advancing next-generation SIK inhibitors with optimized pharmacological properties.

**Figure 9:**
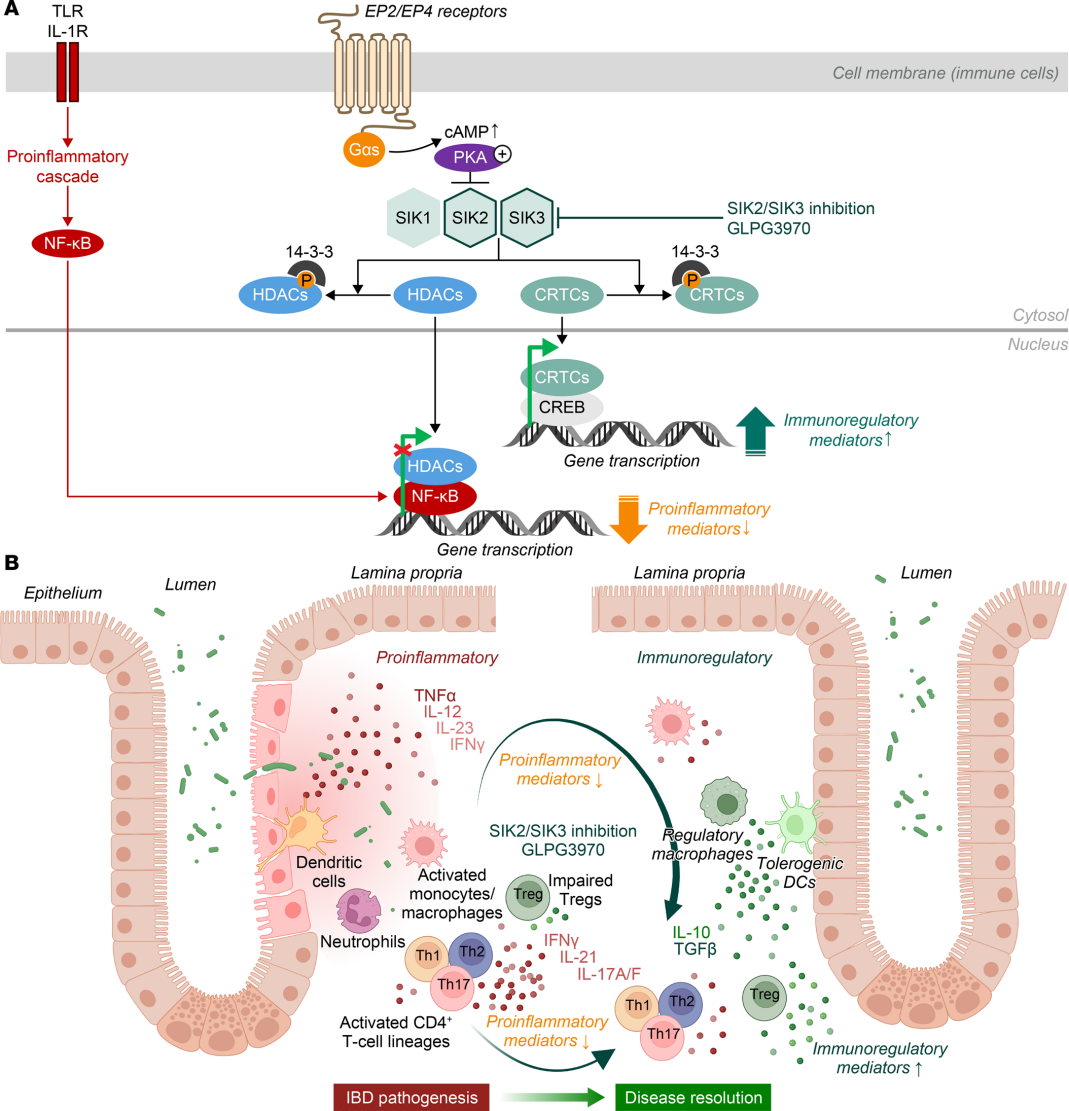
SIK inhibition pathway and proposed mechanism of action of SIK2/SIK3 inhibition in IBD pathogenesis. (**A**) Regulation of cytokine production by SIK2/SIK3 inhibition. GLPG3970 modulates pro- and antiinflammatory cytokine production in TLR- and IL-1R–activated myeloid immune cells. SIK inhibition controls NF-κB– and CREB-regulated gene expression under inflammatory conditions via histone deacetylase (HDAC) and CREB-regulated transcription coactivator (CRTC) substrates. (**B**) Proposed mechanism of action of SIK2/SIK3 inhibition in chronic intestinal inflammation in IBD. GLPG3970 shifts proinflammatory macrophages and DCs toward an immunoregulatory or tolerogenic phenotype, rebalancing pro- and antiinflammatory pathways and cytokine profiles. This promotes Treg function, reduces T effector activity, and supports tissue repair and disease resolution. CREB, cAMP response element–binding protein; EP2, prostaglandin E2 receptor.

We hypothesized that the predominance of proinflammatory over antiinflammatory myeloid cell states in chronic inflammation may result from dysregulated transcriptional control downstream of SIK signaling, which is normally regulated by cAMP/PKA pathways activated by GPCRs (Figure 9A). While this mechanistic framework has been proposed, functional evidence for aberrant SIK activity in autoimmune disease has remained limited, partly because SIK2 and SIK3 are ubiquitously and constitutively expressed in affected tissues. In addition, a lack of selective inhibitors has constrained mechanistic interrogation in human systems. To address these gaps, we developed GLPG3970, a highly selective SIK2/SIK3 inhibitor, and systematically evaluated its capacity to reprogram mononuclear phagocytes from proinflammatory to antiinflammatory states. This was achieved through a translational bridging approach, integrating in vitro assays of primary human immune cells with preclinical models of colitis, PsO, and arthritis, and extending into short signal-detection clinical studies in PsO, UC, and RA patients.

In vitro analyses of GLPG3970 using disease-relevant functional assays revealed a robust immunomodulatory effect across multiple immune cell types. In monocytes, macrophages, and DCs stimulated with LPS, a microbial-derived TLR4 agonist that strongly activates NF-κB signaling, GLPG3970 potently suppressed the secretion of key proinflammatory cytokines, including TNF-α, IL-12p40, and IL-23, as well as chemokines such as MCP-1, MCP-2, GM-CSF, and M-CSF, all of which contribute to the amplification and persistence of chronic inflammation (6, 7, 50). In parallel, GLPG3970 induced CREB-dependent transcription, leading to upregulation of IL-10 and mediators such as THBS1, AREG, VEGF, and G-CSF, which are involved in promoting the resolution of inflammation and supporting tissue repair (7, 51, 52). While IL-10 has well-established immunoregulatory functions in maintaining tissue homeostasis, the roles of THBS1 and VEGF are less clearly defined; THBS1 may facilitate activation of latent TGF-β to drive antiinflammatory responses and tissue repair, whereas macrophage-derived VEGF may support tissue regeneration and restoration of intestinal barrier integrity (53, 54). Beyond its effects in differentiated cells, SIK2/SIK3 inhibition also reprogrammed monocyte-to-DC differentiation toward a stable tolerogenic phenotype. When GLPG3970 was administered during differentiation, it induced sustained IL-10 production and upregulated markers such as ILT4, CD163, CD209, and HLA-DR, while suppressing proinflammatory cytokines including TNF-α, IL-12p40, IL-6, and IL-23 upon LPS stimulation (33–36). Such a shift toward an immunoregulatory phenotype is of particular interest, as tolerogenic DCs can critically shape T cell responses by promoting regulatory rather than effector subsets, thereby contributing to the resolution of chronic inflammation (33, 55). Notably, the IL-23–Th17 axis has emerged as a central pathway in the pathogenesis of PsO and IBD, with IL-23–targeted therapies demonstrating strong clinical efficacy (56).

The immunomodulatory effects of GLPG3970 were not restricted to the myeloid cell compartment. In T cell receptor–activated PBMCs, GLPG3970 suppressed IL-2 and IFN-γ, thereby attenuating signals essential for T cell proliferation, differentiation into T effector lineages, and chronic inflammation (57, 58). IFN-γ is an important amplifier of proinflammatory macrophages (6). Furthermore, in B cells activated via B cell receptor and CD40 engagement, GLPG3970 induced a regulatory phenotype characterized by increased IL-10 production and a transcriptomic profile resembling TLR9-induced regulatory B cells (38). To our knowledge, this represents the first demonstration that selective SIK2/SIK3 inhibition attenuates T cell activation while simultaneously promoting a stable Breg phenotype in human cells. IL-10–producing Bregs play a critical role in controlling immune responses and have been shown to promote mucosal healing in a mouse model of colitis (37, 59). Collectively, these findings position SIK2/SIK3 as key regulators of the balance between inflammatory and immunoregulatory states across innate and adaptive immune cell compartments. While the data suggest broad immunomodulatory potential, further studies are needed to clarify the molecular pathways driving these adaptive immune effects.

To extend our in vitro findings into more complex disease settings, we evaluated the therapeutic potential of GLPG3970 in established murine models of immune-mediated pathology, including colitis, PsO-like skin inflammation, and arthritis. Across all models, GLPG3970 significantly reduced both clinical and histopathological disease activity, whether administered prophylactically or therapeutically, supporting its efficacy in active disease contexts.

In models of chronic intestinal inflammation, GLPG3970 demonstrated consistent activity across multiple colitis paradigms. In the acute dextran sulfate sodium–induced colitis model, prophylactic treatment reduced disease severity and improved histological outcomes (31, 42). These effects were recapitulated in the adoptive T cell transfer and Mdr1a^–/–^ chronic colitis models, both widely used for preclinical evaluation of IBD therapeutics (41, 45). The adoptive transfer model reflects colitis driven by impaired immune tolerance, whereas the Mdr1a^–/–^ model features spontaneous inflammation with epithelial barrier dysfunction and IL-23–Th17 axis activation, closely resembling human IBD based on its responsiveness to anti–IL-12/IL-23 therapies (60). GLPG3970 significantly reduced clinical and histological disease activity in both models, including preservation of goblet cell integrity.

The partial efficacy of GLPG3970 (30 mg/kg twice daily) in the Mdr1a^–/–^ model, relative to an anti–IL-12p40 antibody, likely reflects pharmacokinetic limitations. With a short plasma half-life in mice (0.55 hours), GLPG3970 provides target coverage for about 16 hours per day (31), whereas the antibody ensures continuous cytokine blockade, potentially accounting for its superior efficacy in this setting.

Mechanistic insights into the immunomodulatory effects of GLPG3970 in chronic colitis were obtained through transcriptomic profiling of colonic tissue from the T cell transfer model, which revealed attenuation of NF-κB and JAK-STAT signaling pathways, downregulation of inflammatory monocyte and type 2 DC markers, and upregulation of epithelial cell markers, collectively indicating a shift from a proinflammatory to a pro-resolving tissue state. Cytokine profiling further supported this transition, showing reduced levels of TNF-α and other proinflammatory mediators (IL-1β, IL-12, IL-23, MCP-1, IFN-γ, IL-17, IL-22), alongside increased expression of IL-10, THBS1, and VEGF, consistent with an immunoregulatory and tissue-repairing environment.

Flow cytometric analysis of lamina propria mononuclear cells revealed a reduction in total macrophage and DC numbers, with a phenotypic shift toward IL-22^+^ and M2-like macrophages, decreased inflammatory M1 macrophages, and increased tolerogenic PD-L1^+^/PD-L2^+^ DCs. Although overall T cell infiltration remained unchanged, the Treg/Th17 ratio increased as a result of a selective reduction in Th17 cells. These cellular and molecular changes suggest that GLPG3970 reprograms mucosal mononuclear phagocytes toward an antiinflammatory phenotype, partly via suppression of NF-κB signaling, a key driver of myeloid activation in intestinal inflammation (50). Notably, the monocyte and DC subsets modulated by GLPG3970 are expanded in colonic tissue from patients with UC and have been linked to resistance to anti–TNF-α therapy (61). Interestingly, in keeping with Chebli et al. (47), we identified a modest but significant increase in the percentage of IL-22–expressing macrophages, a finding that may reflect a tissue-reparative phenotype. Given IL-22’s dual role in antimicrobial defense and epithelial regeneration, this macrophage subset could contribute to mucosal healing in chronic inflammation, distinct from the elevated IL-22 levels typically attributed to Th effector cells in colonic tissue (47). This observation is consistent with evidence that IL-22 exerts context-dependent effects in colitis, with IL-22 from Th17/Th22 cells potentially contributing to pathogenic inflammation, in contrast to a reparative role of IL-22^+^ macrophages (62).

Overall, these findings provide robust preclinical support for selective SIK2/SIK3 inhibition as a therapeutic strategy in chronic intestinal inflammation, with observed immune rebalancing potentially supporting epithelial integrity, goblet cell preservation, and mucosal repair, thereby extending prior evidence of SIK inhibition’s antiinflammatory effects in LPS/sepsis and dextran sulfate sodium colitis models (31, 63, 64).

In PsO models, GLPG3970 treatment dose-dependently reduced epidermal thickness in both IL-23– and imiquimod-induced models. Maximal efficacy in the IL-23 model was comparable to that of a TYK2 inhibitor, while in the imiquimod model, GLPG3970 significantly attenuated histopathological inflammation and neutrophilic infiltration. These findings align with the central role of IL-23 in PsO pathogenesis (11) and suggest that GLPG3970-mediated suppression of IL-23 by mononuclear phagocytes contributes to its in vivo efficacy. Concomitant induction of IL-10 by myeloid cells and Bregs may promote immune tolerance through Treg expansion and inhibition of Th17 responses (65–67). The combined effects of IL-23 suppression, IL-10 upregulation, and reduced T cell activation likely drive the therapeutic benefit observed in PsO models.

In the mouse CIA model, GLPG3970 administered after disease onset produced a dose-dependent reduction in clinical scores and disease progression, with statistically significant effects only at the highest dose. Efficacy was comparable with that of the anti–TNF-α comparator. The positive effect of GLPG3970 on bone erosions further indicated its therapeutic potential. As CIA pathogenesis is primarily driven by adaptive immune mechanisms, including T and B cell activation, autoantibody production, and immune complex–mediated inflammation (68), our in vitro observations that myeloid cells are more sensitive to SIK2/SIK3 inhibition than lymphocytes suggest that higher systemic exposures may be required to achieve comparable therapeutic effects in RA, compared with colitis or PsO, where myeloid cells may represent the more dominant pathogenic drivers.

Together, GLPG3970 effectively modulated disease activity across diverse preclinical disease models by reprogramming mononuclear phagocytes toward antiinflammatory and tissue-protective states, with parallel effects on adaptive immunity, providing a mechanistic rationale for advancing selective SIK2/SIK3 inhibition into translational studies for autoimmune and inflammatory diseases, particularly those predominantly driven by myeloid-mediated pathology.

Building on preclinical evidence supporting the immunomodulatory effects of SIK2/SIK3 inhibition, we evaluated GLPG3970 in 3 short, placebo-controlled signal-detection studies in patients with moderately to severely active UC, PsO, or RA. These studies were designed to assess early biological activity and provide insight into the translational potential of SIK2/SIK3 inhibition in human autoimmune disease. To mitigate a potential QTc prolongation signal, daily dosing of GLPG3970 was capped at 400 mg. Pharmacokinetic modeling indicated that exposures in the 350 to 400 mg range could achieve partial clinical efficacy. Across all studies, GLPG3970 was well tolerated. Most treatment-emergent adverse events (TEAEs) were classified as mild or moderate and were considered unrelated to the study drug by investigators, and few TEAEs led treatment discontinuation.

In UC, the primary endpoint of change in the Mayo Clinic Score (a composite measure of stool frequency, rectal bleeding, endoscopic severity, and physician’s global assessment) did not lead to significant differences between GLPG3970 and placebo. However, predefined secondary endpoints with more stringent and objective measures of disease activity, including endoscopic and histological improvements, favored GLPG3970 over placebo. While these measures did not reach statistical significance in this small study, they were considered clinically meaningful and were further supported by post hoc analyses that showed histological improvement correlated with reductions in rectal bleeding with GLPG3970 compared with placebo. Interpretation of these results was limited by baseline imbalances, as patients receiving GLPG3970 had greater disease severity and higher inflammatory burden at baseline compared with patients receiving placebo. In addition, suboptimal drug exposures may have confounded the assessment of clinical efficacy. Indeed, plasma exposures achieved with 400 mg GLPG3970 once daily were markedly lower than the minimal efficacious exposures defined in T cell transfer and Mdr1a^–/–^ murine colitis models, where steady-state concentrations at the lowest effective dose were 3.75-fold and 3.15-fold higher, respectively (Table 1).

In patients with PsO, GLPG3970 treatment significantly reduced disease activity compared with placebo, as measured by PASI, a composite score reflecting affected body surface area and lesion severity (redness, scaling, and thickness). After just 6 weeks, the placebo-corrected improvement in PASI was approximately 33%, similar to early efficacy reported for apremilast or methotrexate (MTX) (69, 70). Interpretation of these results, however, is limited by suboptimal drug exposure, as average steady-state free plasma concentrations in patients were 1.81- to 6.19-fold lower than the minimal efficacious doses observed in IL-23– and imiquimod-induced PsO mouse models, respectively (Table 1).

In contrast, no efficacy signal was observed in patients with RA across primary or secondary endpoints, despite robust effects in the mouse CIA model. This discrepancy is likely due to suboptimal drug exposure, as free plasma levels in RA patients were approximately 11.3-fold lower than the minimal effective dose in the CIA model, representing the largest human-to-mouse exposure gap among the 3 disease settings studied (Table 1). Because RA pathogenesis is driven predominantly by adaptive immune mechanisms rather than myeloid-mediated inflammation, higher drug exposures may be required to achieve meaningful modulation of disease activity.

Taken together, the clinical studies suggest that GLPG3970 was underdosed relative to preclinical benchmarks, particularly in UC and RA, although meaningful efficacy signals were observed in PsO even at sub-therapeutic exposures. This underscores the greater sensitivity of myeloid-driven diseases, such as PsO and colitis, to SIK inhibition compared with lymphocyte-driven conditions like RA, and highlights the importance of achieving adequate systemic exposures, which were limited for GLPG3970 because of QTc concerns at higher doses. Interpretation of these studies is also constrained by their exploratory design, short duration, and limited statistical power, which precluded definitive assessment of efficacy.

Our preclinical and early clinical data support SIK2 and SIK3 as key regulators of myeloid cell function and highlight them as promising therapeutic targets in autoimmune disease. GLPG3970 consistently reprogrammed myeloid cells from proinflammatory to immunoregulatory states, exerting both direct and indirect effects on T and B cell activation, thereby establishing a coherent mechanism that translates from in vitro systems to in vivo models and to early clinical signals in UC and PsO (Figure 9B). Although exposure constraints limited full dose exploration of GLPG3970, these results provide clinical proof of concept for selective SIK2/SIK3 inhibition as a therapeutic strategy in chronic inflammatory diseases. Collectively, our data support continued development of next-generation SIK inhibitors with improved potency, pharmacokinetic properties, and safety pharmacology to achieve optimal systemic exposures and enable comprehensive assessment of their therapeutic and immunomodulatory potential in future clinical trials.

## Methods

The methods for the preclinical in vitro and in vivo studies, as well as further detail regarding clinical study methods, are described in Supplemental Methods.

### Sex as a biological variable

Sex was not considered as a biological variable in the preclinical models or in the clinical studies, and no analyses were done by sex.

### Trial designs

Oral once-daily GLPG3970 treatment was examined for 6 weeks in patients aged 18–65 years across 3 randomized, double-blind, placebo-controlled, multicenter, parallel-group studies in patients with moderately to severely active UC (400 mg GLPG3970; phase II; NCT04577794), PsO (350 mg GLPG3970; phase I; NCT04106297), and RA (400 mg GLPG3970; phase II; NCT04577781).

### Ulcerative colitis

#### Inclusion/exclusion criteria.

Eligible patients had documented UC for at least 3 months of moderate to severe disease activity at screening determined by centrally read endoscopy (modified Endoscopic Subscore [mESS] ≥ 2 or Ulcerative Colitis Endoscopic Index of Severity [UCEIS] ≥ 4) with a minimum disease extent of 15 cm from anal verge, an MCS–stool frequency (SF) subscore of at least 1, and an MCS–rectal bleeding (RB) subscore of at least 1. Patients with a diagnosis of Crohn’s disease or other forms of colitis and those who had complications or previous surgical intervention were excluded.

#### Endpoints and outcome measures.

The primary endpoint was the change from baseline in total MCS (including RB score, mESS, SF score, and patient global assessment) at week 6. Secondary endpoints were the number, incidence, and severity of TEAEs. Additional endpoints presented herein are MCS response, MCS remission, endoscopic response, and histologic-endoscopic mucosal index (HEMI). Details on randomization are described in the supplemental material.

### Psoriasis

#### Inclusion/exclusion criteria.

Eligible patients had moderate to severe plaque PsO (PASI of at least 12) for at least 6 months, a body mass index (BMI) of 18–35 kg/m^2^, and at least 10% of body surface area affected.

#### Endpoints and outcome measures.

The primary endpoint was safety per frequency and severity of TEAEs, serious adverse events, and TEAEs leading to treatment discontinuations. Other endpoints included the percentage change from baseline in the PASI score and a reduction of 50%, 75%, 90%, or 100% in the PASI score relative to baseline. Details on randomization are described in the supplemental material.

### Rheumatoid arthritis

#### Inclusion/exclusion criteria.

Eligible patients had a diagnosis of RA for at least 6 months meeting the 2010 ACR/European Alliance of Associations for Rheumatology (EULAR) criteria, and a DAS28-CRP greater than 3.2 (indicating at least moderate disease). Patients had an inadequate clinical response to MTX, had been receiving MTX for at least 6 months, and had been on a stable dose (10–20 mg/wk) for at least 4 weeks. Patients with a BMI of 18–32 kg/m^2^ were included. Patients had at least 6 swollen joints (evaluated in 66 joints [SJC66]) and at least 8 tender joints (evaluated in 68 joints [TJC68]) at screening and baseline.

#### Endpoints and outcome measures.

The primary endpoint was the change from baseline in DAS28-CRP at week 6. The secondary endpoints were the frequency, incidence, and severity of TEAEs. Details on randomization are described in the supplemental material.

### Statistics

#### Preclinical in vitro and in vivo studies.

All in vitro and in vivo experiments were conducted with independent biological replicates as specified in the respective methods. Data are presented as mean ± SEM, unless stated otherwise. Statistical tests are indicated in the figure legends. For comparisons between 2 groups, 2-tailed Student’s *t* test was used. For multiple-group comparisons, 1-way ANOVA was applied when a single independent variable was tested, and 2-way ANOVA when 2 independent variables were tested, followed by appropriate post hoc multiple-comparison tests (Dunnett’s or Bonferroni’s) or false discovery rate (FDR) correction for multiple testing. Kruskal-Wallis test followed by Dunn’s multiple-comparison test was applied for nonparametric data. For RNA sequencing and gene set enrichment analyses, adjusted *P* values (Benjamini-Hochberg method) were used to determine significance. For correlation analyses, Spearman’s correlation was applied.

Statistical significance was defined as *P* < 0.05 unless otherwise indicated.

#### Clinical studies.

For patients with UC, a sample size of 30 patients (GLPG3970, *n* = 20; placebo, *n* = 10) was planned. For the primary efficacy endpoint, total MCS was analyzed descriptively by group, as actual (observed) values and changes from baseline. Analysis of covariance was used on the total MCS change from baseline to compare treatment groups, with treatment as a fixed effect and the baseline total MCS as a continuous covariate. For total MCS, missing data were imputed using Rubin’s multiple imputation. For analyses of binary endpoints (MCS response, MCS remission, endoscopic response, HEMI), nonresponder imputation was used for missing data imputation. Efficacy analyses were performed on the full analysis set. For the safety endpoint, all safety data collected on or after the first dosing up to the last contact after the last dosing were summarized by treatment group according to the actual treatment received. Clinical safety was evaluated by assessment of adverse events (AEs), laboratory assessments, vital signs, 12-lead electrocardiograms (ECGs), and physical examinations. All safety analyses were performed using the safety analysis set.

For patients with PsO, a sample size of 25 patients (GLPG3970, *n* = 15; placebo, *n* = 10) was planned. The exploratory treatment difference between GLPG3970 and placebo was assessed by means of mixed-effects repeated-measures model-derived LS mean change from baseline. All safety data collected on or after the first dose of GLPG3970 or placebo up to the last follow-up visit after the last dose of GLPG3970 or placebo, unless specified otherwise, were summarized by treatment group according to the actual treatment received. All safety analyses were performed using the safety analysis set. Safety data from patients with moderately to severely active PsO in the first-in-human study were not pooled. Clinical safety was addressed by assessment of AEs, laboratory assessments, physical examinations, vital signs, and 12-lead ECGs.

For patients with RA, a sample size of 25 patients (GLPG3970, *n* = 15; placebo, *n* = 10) was planned. For the primary efficacy endpoint, disease assessment score, considering 28 joint counts, and C-reactive protein (DAS28-CRP) data were analyzed using descriptive statistics as actual (observed) values and changes from baseline. A mixed model for repeated measures was used to derive LS means (SE) to compare between GLPG3970 and placebo based on DAS28-CRP changes from baseline and treatment difference at week 6. All safety data collected on or after the first dosing and up to the last contact after the last dose of treatment, unless specified otherwise, were summarized by treatment group according to the treatment received. Clinical safety was addressed by assessment of AEs, laboratory assessments, vital signs, 12-lead ECGs, and physical examinations.

### Study approval

Mouse studies were performed according to ethical guidelines approved by the Animal Institutional Care and Use Committee of Galapagos controlled by the French Authorities (Agreement 93-063-06, Direction Départementale de la Protection des Populations, Seine-Saint-Denis).

Human studies (UC, NCT04577794; PsO, NCT04106297; RA, NCT04577781) ) were approved by an institutional review board or ethics committee at each study site (see Supplemental Materials) and were conducted according to the International Council for Harmonisation for Good Clinical Practice Guidelines, the Declaration of Helsinki, and local laws and regulations. Patients provided written informed consent before study enrollment.

### Data availability

Data values for figures are provided in the Supporting Data Values file.

Additional data obtained from preclinical Galapagos-sponsored research are unavailable, to protect intellectual property rights. Galapagos is committed to publish sponsored clinical trial results in peer-reviewed scientific journals as required by law and/or regulations. Deidentified participant data from this study, along with relevant documentation (analysis datasets, data dictionary, and statistical analysis plan), are available upon reasonable request to qualified researchers. All requests will be reviewed by an internal data review committee which will validate access only if the request meets the criteria outlined in this policy and based on the committee’s discretionary decision. Data will be shared after publication of the primary manuscript and upon execution of a data sharing agreement. Data requests are submitted via dpo@glpg.com. Data are made available for noncommercial research purposes only.

## Author contributions

SDV, ND, APF, SL, DM, CD, CJ, AC, AL, NVO, DA, M Constant, AGH, WOB, and GD contributed to the conceptualization, design, or methodology of this study. SDV, SB, ND, APF, SL, DM, CD, CJ, AC, DA, CP, M Colli, TVE, TH, M Constant, AGH, RB, and WOB contributed to the acquisition or analysis of the data. SDV, ND, APF, SL, DM, CD, CJ, AC, CP, M Colli, TVE, TH, M Constant, and RB contributed to the visualization or validation of the data. SDV, SB, AL, NVO, M Colli, TVE, RB, and WOB contributed to the interpretation of the data. All authors contributed to manuscript development and approved the final version for submission.

## Funding support

- Galapagos NV (Mechelen, Belgium).

## Supplementary Material

Supplemental data

Supporting data values

## Figures and Tables

**Table 1:**
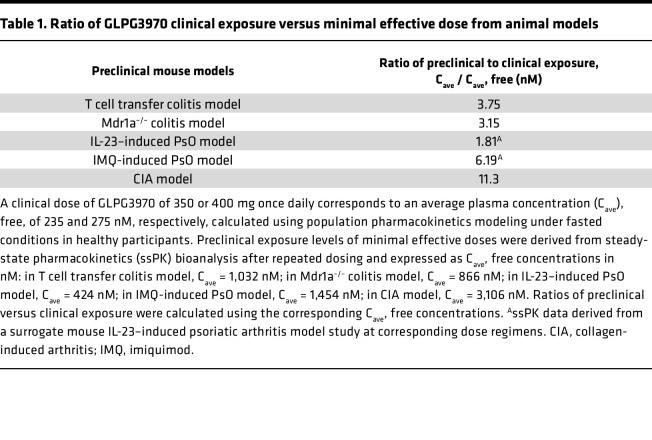
Ratio of GLPG3970 clinical exposure versus minimal effective dose from animal models

## Version 1 02/09/2026

Electronic publication
