# Supplementary material for: Selective SIK2/SIK3 inhibition reprograms pro- and antiinflammatory pathways in myeloid cells, improving autoimmune disease outcomes
Source: JCI Insight. 2026 Feb 9;11(3):e171776. doi: 10.1172/jci.insight.171776 (PMC12893107; doi:10.1172/jci.insight.171776)
Supplement: Supplemental data [file jciinsight-11-171776-s280.pdf]

Selective SIK2/SIK3 Inhibition Reprograms Pro- and Anti-Inflammatory Pathways in Myeloid Cells, Improving Autoimmune Disease Outcomes

Authored by: Steve De Vos,[1] Nicolas Desroy,[2] Susan Bellaire,[3] Anna Pereira Fernandes,[1] Stéphanie Lavazais,[2] Didier Merciris,[2] Carole Delachaume,[2] Catherine Jagerschmidt,[2] Adrien Cosson,[2] Angela Lazaryan,[2] Nancy Van Osselaer,[1] David Amantini,[2] Christophe Peixoto,[2] Maikel Colli,[1] Thomas Van Eeckhoutte ,[1] Tiina Hakonen,[1] Magali Constant,[1] Alberto Garcia Hernandez,[1] Rahul Barron,[4] Geert D'Haens[*,5] Wulf Otto Böcher[*,4]

*Geert D'Haens and Wulf Otto Böcher are co-senior authors.

1. Galapagos NV, Mechelen, Belgium
2. Galapagos SASU, Romainville, France
3. Galapagos BV, Leiden, The Netherlands
4. Galapagos GmbH, Basel, Switzerland
5. Department of Gastroenterology and Hepatology, Amsterdam University Medical Centres, Amsterdam, Netherlands

Corresponding author: Steve De Vos; Galapagos NV Generaal De Wittelaan L11 A3 2800 Mechelen, Belgium; Telephone: +32 499 74 57 32; email: steve_de_vos@telenet.be

Supplementary Material

Materials and Methods

Isolation of $CD14^+$ Monocytes

Human peripheral blood mononuclear cells (PBMCs) were isolated from healthy donor blood (Red Cross Vlaanderen, Mechelen, Belgium) using Lymphoprep (Axis-Shield). Granulocytes were depleted (StemCell Technologies), residual erythrocytes lysed (BioLegend), and $CD14^+$ monocytes purified using antibody-conjugated magnetic beads (Miltenyi Biotec).

Primary Monocyte Assay

$CD14^+$ monocytes were seeded in 96-well plates, pre-incubated for 1 h with serial dilutions of test compound, then stimulated with lipopolysaccharide (LPS; 100 ng/mL; Sigma-Aldrich). Supernatants were collected for cytokine analysis (ELISA single-plex: R&D Systems, BD Pharmingen; multiplex: Meso Scale Discovery) at 4 h (IL-10, TNFα) or 24 h (other analytes).

Monocyte-Derived Macrophage (MdM) and Monocyte-Derived Dendritic Cell (MoDC) Assays

CD14+ monocytes were for 12 days with macrophage colony stimulating factor (M-CSF; 100 ng/mL; PeproTech) for MdMs, or with granulocyte macrophage colony stimulating factor (GM-CSF; 50 ng/mL; PeproTech) and IL-4 (35 ng/mL; R&D Systems) for MoDCs. Differentiated MdMs and MoDCs were pre-incubated with compound for 1 h then stimulated with LPS (100 ng/mL). Supernatants were collected at 4 h (IL-10) or 24 h (other cytokines) and analyzed by ELISA.

Tolerogenic Dendritic Cell (tolDC) Assay

$CD14^+$ monocytes were seeded in 24-well plates (5×10^5 cells/well in RPMI-1640 with 10% fetal bovine serum [FBS] and 1% penicillin/streptomycin) and differentiated for 5 days with GM-CSF and IL-4 (R&D Systems) in the presence of compound. On day 6, cells were reseeded in fresh medium without compound and stimulated with LPS (100 ng/mL). Supernatant cytokines were quantified after 4 h (AlphaLISA, Revvity). For DC phenotyping, cells were stained with LIVE/DEAD Yellow (Thermo Fisher) and the following antibodies: CD14 (M5E2, PerCP-Cy5.5; BD), CD163 (GHI/61, BV711; BD), CD209 (9E9A8, APC; BioLegend), ILT4 (42D1, PE; BioLegend), CD11c (Bu15, PE-Cy7; BioLegend), and HLA-DR (LN3, APC-Cy7; Thermo Fisher). Data were acquired on LSRFortessa X-20 (BD) and analyzed in FlowJo v10.8. Cells were gated on forward/side scatter to exclude debris, doublets were removed, and viability was assessed with LIVE/DEAD dye. Live cells were further gated on $CD14^+CD11c^+$ expression prior to phenotypic analysis.

Primary Human T Cell Activation Assay

PBMCs were plated on anti-CD3–coated plates (3 μg/mL), pre-incubated with compound for 1 h, and stimulated with anti-CD28 (1 μg/mL). Supernatants were collected after 24 h for IL-2 and interferon γ (IFNγ) quantification (ELISA).

Primary Human Tonsillar B Cell Activation Assay

Primary human tonsillar mononuclear cells (AZ Sint-Maarten Hospital, Mechelen, Belgium) were processed using a B cell isolation kit II (Miltenyi Biotec). Purified B cells (10^6 cells/mL) were stimulated with CD40L (1 µg/mL) + enhancer and anti-IgG/IgM (5 µg/mL) in the presence of test compound. IL-10 in supernatant was quantified after 72 h (ELISA).

Preclinical Models

Mice

Mdr1a$^{-/-}$, SCID, BALB/c and DBA/1J mice (Janvier, Taconic Biosciences) were housed under specific pathogen free conditions at 22°C, 12 h light/dark, with food/water ad libitum.

T Cell Transfer Colitis Model

Female severe combined immunodeficient (SCID) mice received intraperitoneal injection of 0.2 mL containing 1.5×10^6 $CD4^+CD25^-$ T cells on day 1. Sham mice received medium alone. On day 15, mice (n=12/group) were randomized and treated orally twice daily with GLPG3970 (10 or 30 mg/kg in vehicle [0.5% Solutol/methylcellulose 2%/98%]), or vehicle; abatacept was used as positive control and administered intraperitoneally 3 times/week. Disease activity index (DAI; combined score of body weight, stool consistency and fecal blood; scored 0–4 each) was assessed twice weekly. Steady state PK sampling (0, 0.25, 1, and 5 hours) was performed on day 21 and quantified by liquid chromatography-mass spectrometry (LC-MS). At day 53, colons were collected for histology, gene expression, and protein analyses 1.25 h post-dosing.

Mdr1a Colitis Model

Female Mdr1a$^{-/-}$ mice (n=10/group) were orally inoculated with *Helicobacter bilis* (ATCC 51360; 10^7 cfu/mouse, on days 1 and 7)(1). GLPG3970 (10 or 30 mg/kg in vehicle) was administered orally twice daily from days 1–38. Positive control: anti-IL-12/23p40 antibody (25 mg/kg, intraperitoneal, weekly). FVB female wild-type mice (receiving *Helicobacter bilis*) served as negative controls. DAI was assessed twice weekly; colons were harvested on day 38 for histology 1.25 h post-dosing.

Histology Analysis

Colons were formalin-fixed, paraffin-embedded, and periodic acid Schiff-stained. Mouse Colitis Histology Index (MCHI) was scored (0–3 each for inflammatory infiltrate, goblet cell loss, crypt hyperplasia, crypt density, muscle thickness, submucosal infiltration, ulcerations, and crypt abscesses).

Transcriptomic Analysis

Colon RNA was extracted (RNeasy, Qiagen). Poly(A) capture was used to enrich for mRNA and long non-coding RNA; libraries were multiplexed and sequenced (NovaSeq 6000, Illumina). Reads were pseudoaligned to hg38 (Ensembl v105, primary assembly) with Kallisto v0.43.1. Differentially

expressed gene (DEG) counts were analyzed with DESeq2 v1.32.0 (R), normalized for sequencing depth/variability, and fit with a negative binomial generalized linear model; Wald test with Benjamini–Hochberg correction was applied to define DEGs (FDR < 0.05, |log2FC| > 1). PCA was performed on the top 500 variable genes after variance-stabilizing transformation. Fast gene set enrichment analysis (10,000 permutations) was applied to ranked gene lists against the Molecular Signature Database hallmark genes, KEGG, Reactome, and a Breg meta-signature (2). Cell type enrichment used published single-cell RNA-seq data from healthy individuals and patients with UC (3); marker genes (log2FC > 1, adj p < 0.01 vs other cell types) were mapped to mouse orthologues. Only adjusted p < 0.05 sets were considered significant. Heatmaps were generated from normalized counts averaged per condition and Z-score standardized.

Protein Analysis of Mouse Colons

Colon lysates were prepared in T-Per *lysis* buffer (Thermo Scientific) with phosphatase/protease inhibitors (Thermo Scientific, Roche) *and p*rotein was quantified (Coomassie, Thermo Scientific). Cytokines were measured by AlphaLISA (TNFα; PerkinElmer), ELISA (IL-10, VEGF, R&D Systems; THBS1, Lifespan Bio), and multiplex (ProcartaPlex Mouse Immune Monitoring 48plex, Invitrogen). Data were normalized to total protein (pg/mg).

Immunophenotyping of Lamina Propria Mononuclear Cells (LPMCs)

Colons were digested with Lamina Propria Dissociation Kit (Miltenyi). LPMCs were stained with Viobility 405/520 Fixable Dye (Miltenyi), treated with Mouse Fc-block (BD Pharmingen), and antibody stained for surface/intracellular markers. For intracellular markers, LPMCs were fixed/permeabilized using Transcription Factor Staining Buffer Set (Miltenyi Biotec). For cytokine detection, LPMCs were stimulated for 4 h with phorbol 12-myristate 13-acetate and ionomycin in brefeldin A (Sigma Aldrich). Antibodies to asses LPMC phenotype included: CD3 (17A2, AF700/PE-Cy7; BD), CD4 (RM4-5, BV605; BD), CD11c (REA754, PE-Vio770; Miltenyi), CD25 (PC61.5, PE-Cy7; eBioscience), CD45 (30-F11, APC-eFluor780/BV605; eBioscience/BD), CD64 (X54-5/7.1, PE/PE-Cy7; BioLegend), CX3CR1 (SA011F11, AF488; BioLegend), F4/80 (BM8, BV605/PE-Cy7; BioLegend/eBioscience), CD206 (C068C2, AF700; BioLegend), MHC II (REA813, APC-Vio770; Miltenyi), PD-L1 (10F.9G2, APC; BioLegend), PD-L2 (MIH37, PE-Vio770; Miltenyi), Foxp3 (150D/E4, eFluor660; eBioscience), IL-17A (TC11-18H10, PE; BD), IL-22 (1H8PWSR, PE; eBioscience), iNOS (CXNFT, PerCP-eFluor710; eBioscience). Data were acquired on BD LSR Fortessa or BD Lyric and analyzed using FlowJo software.

IL-23-Induced Skin Psoriasis Model

Female BALB/cJ mice were anesthetized and right ears were shaved. Right ears received daily intradermal injections of murine IL-23 (1 µg in 20 µL PBS/0.1%BSA) or PBS/BSA vehicle for 4 days (10 mice/group). GLPG3970 (10, 30, 60 mg/kg, oral, twice-daily), TYK2 inhibitor (rac-37, 30 mg/kg, positive control, oral, once daily) (4), or vehicle (0.5% MC) were administered from days 1 to 5, 1 h

before receiving IL-23. Ear thickness was measured daily using an electronic gauge. On day 5, mice were sacrificed.

Imiquimod (IMQ)-Induced Psoriasis Model

Female BALB/cJ mice were anesthetized and ears were shaved. Ears were treated daily with 5% IMQ cream (30 mg; ~1.5 mg active compound) for 4 days (10 mice/group). GLPG3970 (10, 30, 60 mg/kg, oral, twice daily) or methotrexate (7.5 mg/kg, intraperitoneal, daily; positive control) were administered. Sham mice received petroleum jelly. Ear thickness was measured on days 1 and 5. Mice were sacrificed 2 h post-dose on day 5. Ears were collected for histology. Neutrophil infiltration in ear tissues was quantified by IHC staining, measured as the percentage of NIMP-R14–positive area.

Collagen-Induced Arthritis (CIA)Model

Male DBA/1J mice tails received intradermal with type II collagen (1 mg/mL) and complete Freund's adjuvant on day 1 to induce arthritis, boosted with incomplete Freund's adjuvant/ type II collagen (1 mg/mL) on day 22. On day 32, mice (n=10/group; mean score ~2.5) were randomized to receive vehicle (0.5% MC), etanercept (10 mg/kg, intraperitoneal, 3x/w), or GLPG3970 (10, 30, 60 mg/kg, oral, twice daily) until day 47. Arthritis scores (0–4/paw, maximum score of 16) and body weight were monitored daily (5). Blood was collected on day 42 for plasma PK (LC-MS). On day 47, mice were sacrificed and paws collected for Larsen score (bone erosion) and histology The hind paws of each animal were imaged by X-ray, and the severity of bone erosion was scored using a radiological scale (0–5 per paw, with the mean of both paws used for analysis) (6).

Clinical Studies

Ulcerative Colitis

Randomization: Patients were randomized 2:1 to receive oral, once-daily GLPG3970 400 mg or placebo for 6 weeks.

Psoriasis

Randomization: Patients with moderately to severely active PsO were in the study for approximately 11 weeks (screening to follow-up [FU] visit) and were randomized 3:2 to receive once-daily GLPG3970 350 mg or placebo for 6 weeks.

Rheumatoid Arthritis

Randomization: Patients were in the study for up to 11 weeks (screening to FU) and were randomized to 3:2 to receive oral, once-daily GLPG3970 400 mg or placebo for 6 weeks.

Results

Clinical Studies

Demographic and Clinical Characteristics at Baseline

In the UC clinical study, demographics were generally similar between the GLPG3970 400 mg and the placebo groups (**Supplementary Table 1**). Most patients were men (GLPG3970 400 mg: 76.2%; placebo: 90.0%) and all patients were white. At baseline, the mean duration of UC was 7.1 years in the GLPG3970 400 mg group and 6.3 years in the placebo group. Imbalances were reported between the treatment groups for mean Robarts Histologic Index (RHI) values, and in the proportion of patients with Nancy score Grades 1 to 4 and a high Geboes score of 5 across the rectum and sigmoid colon.

In the PsO clinical study, all patients were white and over half of the patients (n = 14, 53.8%) were men (**Supplementary Table 3**). Demographic data were comparable between the treatment groups.

In the RA clinical study, all patients were white and 67.9% were women. At baseline, the mean duration of RA was 7.99 years in the GLPG3970 400 mg group and 7.20 years in the placebo group. There were no clinically meaningful differences in baseline disease characteristics between the two treatment groups (**Supplementary Table 4**).

Safety Outcomes

In the UC clinical study, one patient discontinued treatment with GLPG3970 and the study owing to a treatment-emergent adverse event (TEAE) of a positive severe-acute-respiratory-syndrome-related coronavirus 2 (SARS-CoV-2) test and one placebo-treated patient discontinued the study owing to a non-treatment-emergent AE. Most TEAEs were considered unrelated to study treatment by the investigator (**Supplementary Table 2**). All TEAEs were either resolved or resolving at the early discontinuation (ED) or last FU visit, with the exception of increased lipase levels, decreased blood

lactate dehydrogenase levels, and rash (reported in one patient each). Abnormalities in laboratory parameters considered clinically significant and reported as TEAEs by the investigator were increased lipase levels (n = 3) and increased amylase levels (n = 2), and decreased blood lactate dehydrogenase levels, hyperkalemia, and decreased lymphocyte count (reported in one patient each). There were no clinically significant changes observed in individual patients for vital signs or ECG parameters, except for pyrexia in a patient treated with GLPG3970 that was considered unlikely to be related to study treatment by the investigator.

In the PsO clinical study, the incidence of TEAEs was comparable between the GLPG3970 350 mg group and placebo groups (**Supplementary Table 2**). In the GLPG3970 group, two patients discontinued the study due to a TEAE of SARS-CoV-2 and a TEAE of pruritus. In the placebo group, one patient discontinued the study owing to two TEAEs: PsO and psoriatic arthropathy. Eight treatment-emergent abnormalities in safety parameters (increases in amylase, lipase, alanine aminotransferase [ALT], and aspartate aminotransferase [AST] based on baseline levels) were reported in five patients with PsO as TEAEs by the investigator. No other abnormalities in laboratory, vital signs, ECG, or physical examination parameters were considered clinically significant by the investigator.

In the RA clinical study, six patients (37.5%) in the GLPG3970 group and two patients (16.7%) in the placebo group experienced at least one TEAE during the study (**Supplementary Table 2**). Two patients in the GLPG3970 group discontinued study treatment and the study due to TEAEs; one patient owing to increased ALT levels of Grade 2 and one patient owing to a positive SARS-CoV-2 test. In the GLPG3970 group, there were three patients with ongoing TEAEs (one patient with increased ALT levels, one patient with increased lipase levels, and one patient with cholelithiasis and hepatitis) at the ED or last FU visit, and one patient with a resolving TEAE (positive SARS-CoV-2 test) at the ED visit. TEAEs resolved in all other GLPG3970-treated patients by the ED or last FU visit. In the placebo group, all TEAEs resolved in all patients by the last FU visit, except for one patient with an ongoing TEAE (increased lipase levels) at the last FU visit. All treatment-related TEAEs were isolated cases except for dyspepsia that was reported in two GLPG3970-treated patients. Overall, there were no notable observations in vital signs or ECG parameters in patients with RA.

Disclosure of use of generative artificial intelligence

During the preparation of this work, the authors used Chat Generative Pre-Trained Transformer (ChatGPT-5; OpenAI) to reduce wordcount. After using this tool, the authors reviewed and edited the content as needed and take full responsibility for the content of the publication.

Supplementary References

1. Shomer NH, et al. Helicobacter bilis-induced inflammatory bowel disease in scid mice with defined flora. *Infect Immun.* 1997;65(11):4858–64.
2. Dubois F, et al. Transcriptional meta-analysis of regulatory B cells. *Eur J Immunol.* 2020;50(11):1757–69.
3. Smillie CS, et al. Intra- and Inter-cellular Rewiring of the Human Colon during Ulcerative Colitis. *Cell.* 2019;178(3):714–30 e22.
4. Liang J, et al. Lead optimization of a 4-aminopyridine benzamide scaffold to identify potent, selective, and orally bioavailable TYK2 inhibitors. *J Med Chem.* 2013;56(11):4521–36.
5. Nishida K, et al. Histone deacetylase inhibitor suppression of autoantibody-mediated arthritis in mice via regulation of p16INK4a and p21(WAF1/Cip1) expression. *Arthritis Rheum.* 2004;50(10):3365-76.
6. Larsen A. How to apply Larsen score in evaluating radiographs of rheumatoid arthritis in long-term studies. *J Rheumatol.* 1995;22(10):1974–5.

Supplementary Figures and Tables

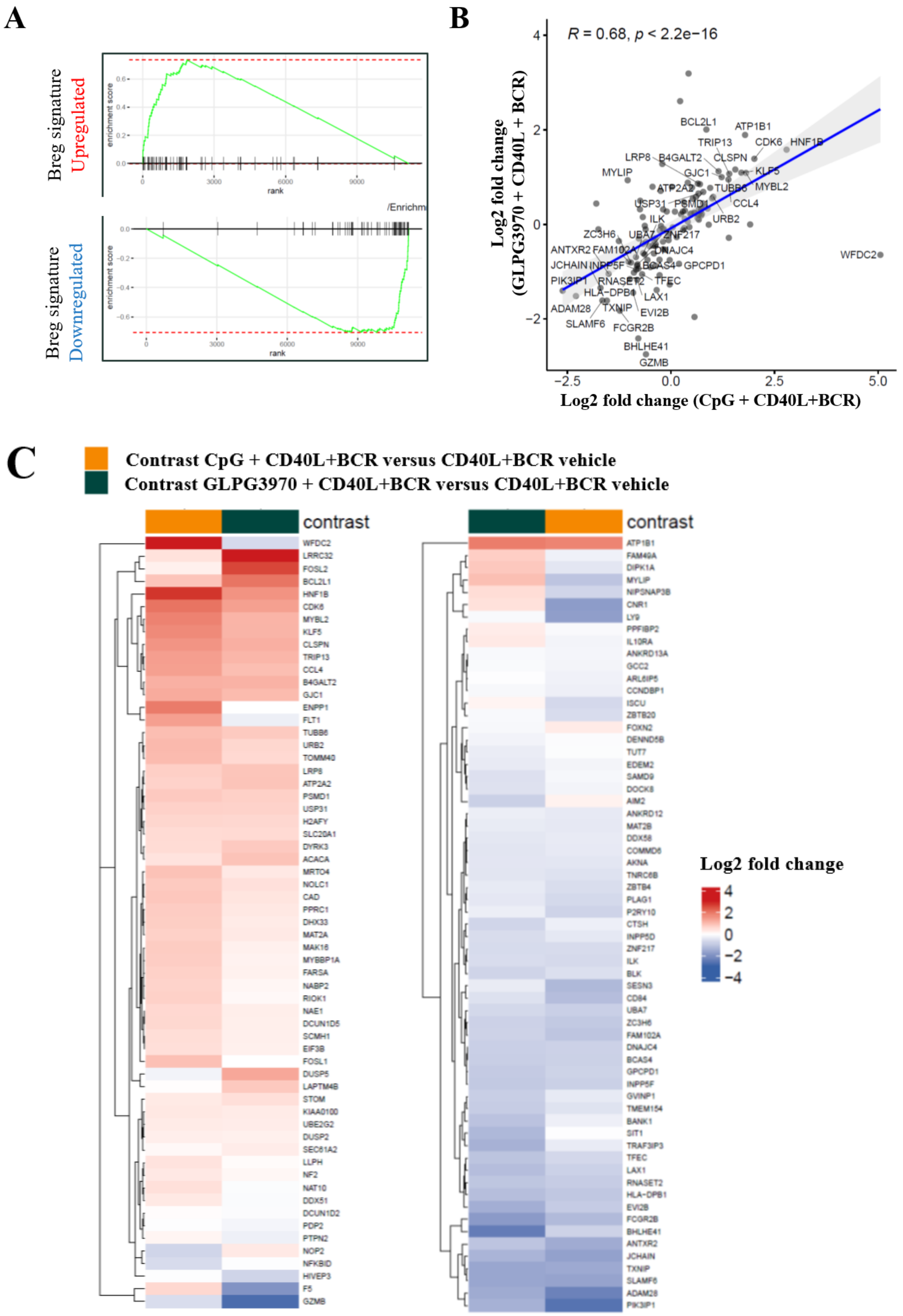

Supplementary Figure 1. GLPG3970 induces a Breg-like transcriptomic signature

(A) Gene set enrichment analysis of Breg signatures according to Dubois et al., 2020 (2). Up- and downregulated Breg gene sets are shown separately for CD40L/anti-BCR-stimulated tonsillar B cells treated with CpG (Breg inducer) compared with vehicle-treated controls.
(B) Spearman correlation of log_2 fold changes for Breg signature genes identified by RNA sequencing in CD40L/anti-BCR-stimulated tonsillar B cells treated with GLPG3970 versus CpG-treated B cells.
(C) Heatmap showing log_2 fold-change of individual genes (vertical axis) upregulated (left) or downregulated (right) in the Breg signature. Horizontal axes show contrasts for CpG-treated versus vehicle-treated B cells (orange) and GLPG3970-treated versus vehicle-treated B cells (green).

BCR, B cell receptor; Breg, regulatory B cell; RNAseq, RNA sequencing.

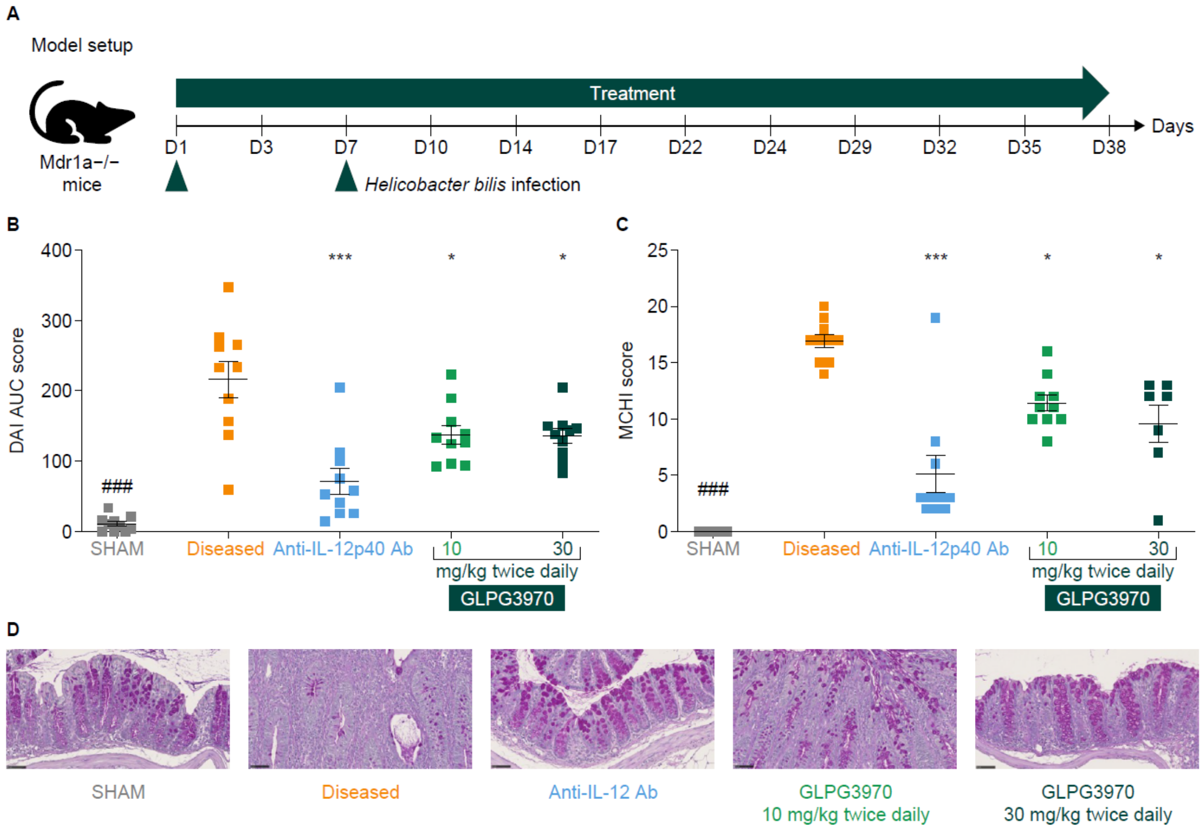

Supplementary Figure 2. SIK2/SIK3 inhibition attenuates disease in the Mdr1a$^{-/-}$ colitis model

(A) Mdr1a^{-}/$^{-}$ colitis mouse model. Mdr1a^{-}/$^{-}$ mice, which lack the transmembrane efflux pump p-glycoprotein, develop spontaneous colitis. Oral infection with *Helicobacter bilis* (10^7 cfu/mouse on days 1 and 7) accelerates disease onset and increases inflammation severity. Animals with disease were treated with GLPG3970 (10 or 30 mg/kg orally twice daily) from day 1 to day 38. An IL-12p40 antibody (25 mg/kg, intraperitoneally, once per week) was included as an active comparator.

(B) AUC of DAI (a composite score of body weight loss, stool consistency, and rectal bleeding) was recorded every 3 days.

(C) MCHI. Data are presented as mean ± SEM (n = 7–10 mice/group).

(D) Representative PAS-stained colon sections. Scale bar: 100 µm.

Statistical analyses: Significance of GLPG3970 versus diseased vehicle was assessed by one-way ANOVA with Dunnett's multiple comparisons test. $^{###}p < 0.001$; $^{*}p < 0.05$; $^{***}p < 0.001$.

Ab, antibody; ANOVA, analysis of variance; AUC, area under the curve; cfu, colony-forming unit; D, day; DAI, Disease Activity Index; IL, interleukin; MCHI, Mouse Colitis Histology Index; Mdr1a^{-}/$^{-}$, multidrug resistance targeted mutation; PAS, periodic acid–Schiff; SEM, standard error of the mean; SIK, salt-inducible kinase.

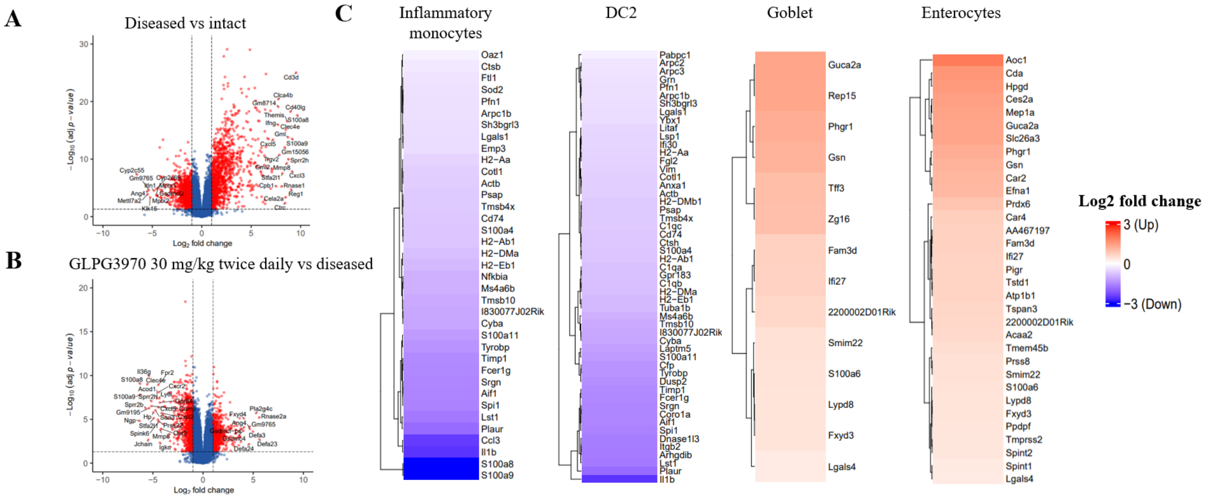

Supplementary Figure 3. T cell colon tissue RNA sequencing (RNAseq)

(A) Volcano plot of DEGs in animals with colitis versus healthy controls.

(B) Volcano plot of DEGs in animals with colitis treated with GLPG3970 (30 mg/kg, twice daily) versus healthy controls. Each gene is plotted as $\log_2$ fold change (x-axis) versus $-\log_{10}$(adjusted p-value) (y-axis). The horizontal dashed line represents $-\log_{10}$(adjusted p-value) > 1.3 (adjusted $p < 0.05$), and the vertical dashed lines indicate $\log_2$ fold change > 1.

(C) Log_2 fold changes of leading-edge genes, representing marker genes contributing most to the enrichment of relevant cell types shown in Figure 4F.

DEG, differentially expressed gene; RNAseq, RNA sequencing.

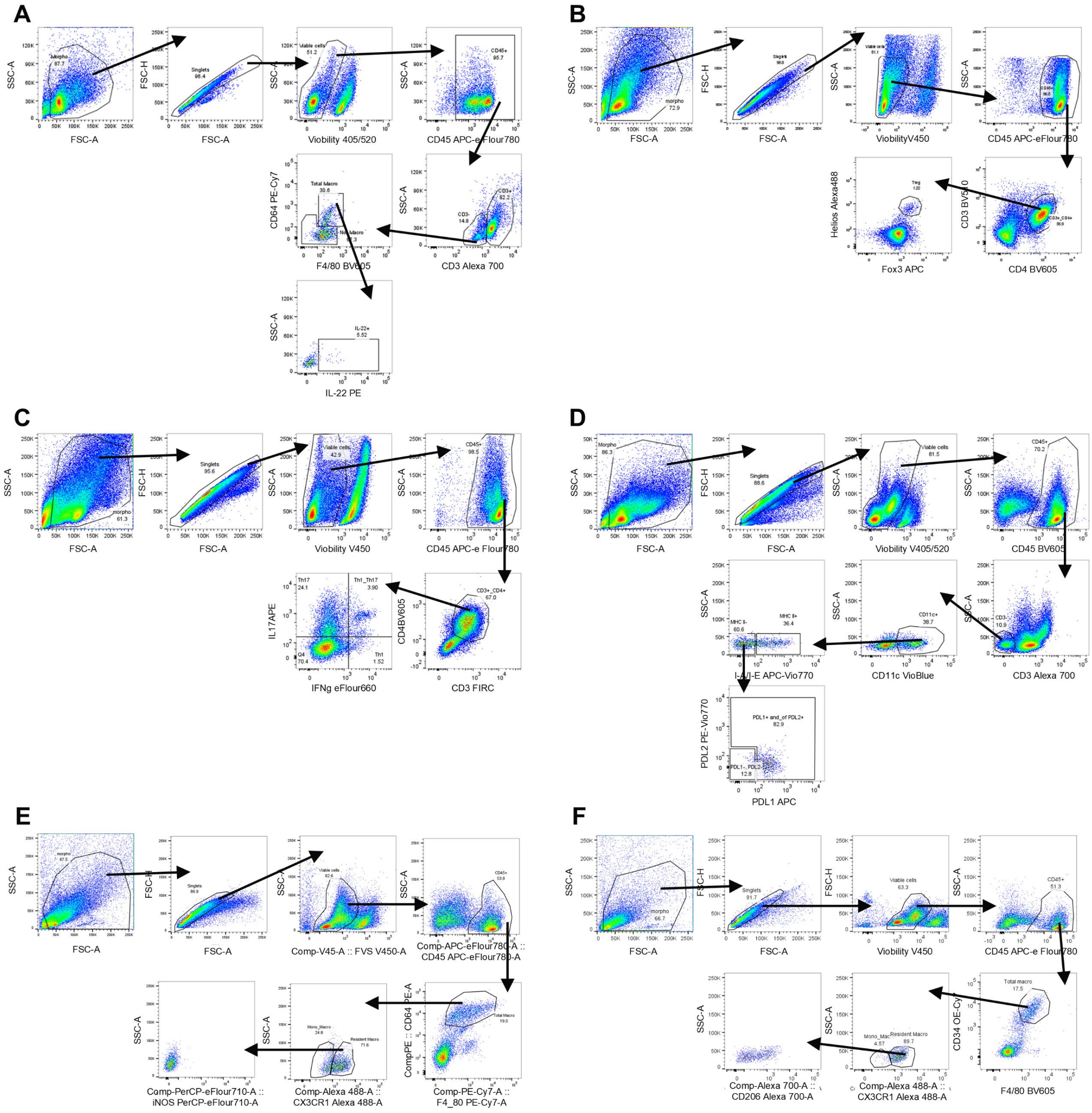

Supplementary Figure 4. Gating strategies used for flow cytometry analyses of LPMCs

Representative gating strategies for flow cytometry analysis of lamina propria mononuclear cells isolated from one mouse in the T cell transfer colitis model. Singlets and viable cells were selected for all analyses.

(A) Gating strategy for assessment of IL-22 expression (MFI) in intestinal macrophages, identified by co-expression of CD64 and F4/80.

(B) Identification and quantification of regulatory T cells co-expressing Foxp3 and Helios.

(C) Quantification of Th17 cells expressing IL-17A.

(D) Identification of tolerogenic DCs, defined as $CD3^{-}CD11c^{+}MHC\ II^{-}PD\text{-}L1^{+}$ and/or $PD\text{-}L2^{+}$.

(E) Quantification of iNOS expression (MFI) in resident macrophages, identified by co-expression of CD64, F4/80 and CX3CR1.

(F) Quantification of CD206 expression (MFI) in resident macrophages, identified by co-expression of CD64, F4/80 and CX3CR1.

DC, dendritic cell; IL, interleukin; MFI, median fluorescence intensity, MFI; Th, T helper cell

A

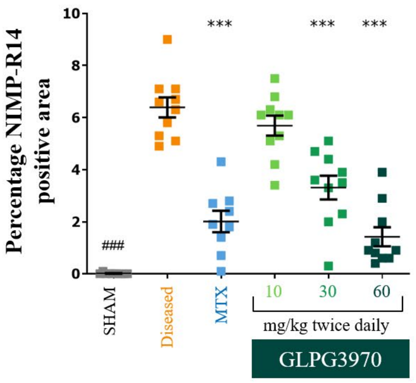

B

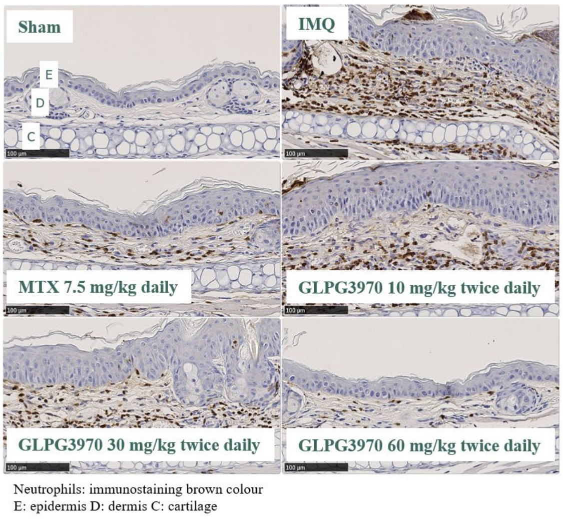

Supplementary Figure 5. GLPG3970 suppresses neutrophil infiltration in the skin

(A) Neutrophil infiltration in the IMQ-induced psoriasis-like skin inflammation mouse model treated with GLPG3970 (10, 30, and 60 mg/kg twice daily). Neutrophil levels were quantified by IHC staining as the percentage of NIMP-R14–positive area.

(B) Representative histological images showing immunostaining of neutrophils (brown) in the epidermis (E), dermis (D), and cartilage (C).

Statistical analyses: Significance versus disease vehicle was assessed by one-way ANOVA with Dunnett's multiple comparisons test. $^{###}p < 0.001$; $^{***}p < 0.001$.

ANOVA, analysis of variance; IHC, immunohistochemistry; IMQ, imiquimod; MTX, methotrexate; NIMP, anti-neutrophil antibody.

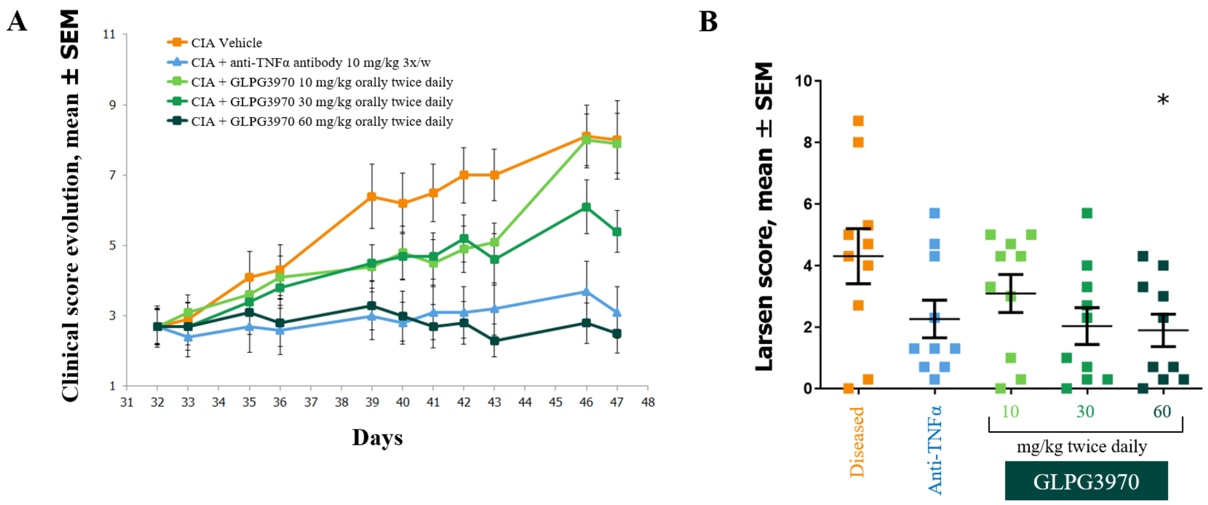

Supplementary Figure 6. GLPG3970 attenuates arthritis and bone erosion

(A) Clinical arthritis scores in the CIA mouse model.

(B) Bone erosion in mouse paws assessed using the Larsen score. Data are presented as mean ± SEM (n = 10 mice/group).

Statistical analyses: Significance versus disease vehicle was assessed by one-way ANOVA with Dunnett's multiple comparisons test. *$p < 0.05$.

ANOVA, analysis of variance; CIA, collagen-induced arthritis; SEM, standard error of the mean; TNFα, tumor necrosis factor alpha.

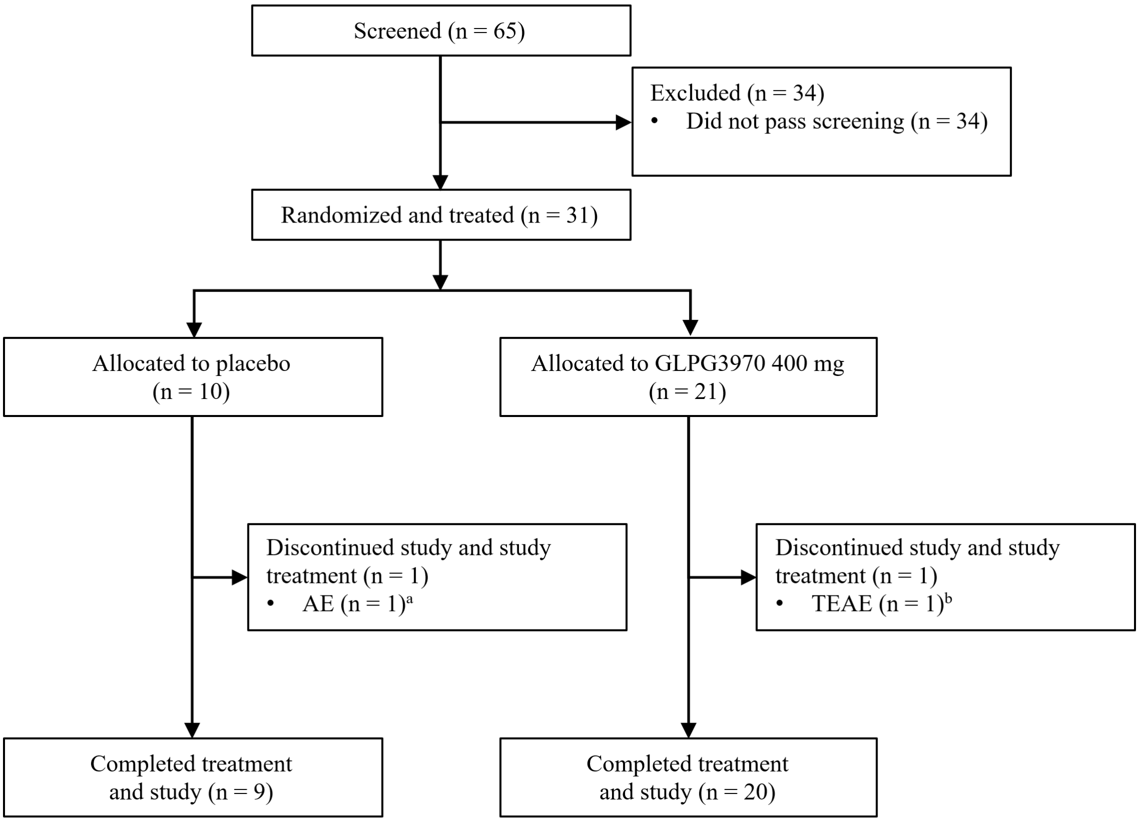

Supplementary Figure 7. Trial profile for UC

[a]One patient discontinued placebo treatment due to a non-treatment-emergent AE as the patient had a prolonged electrocardiogram corrected QT interval. [b]One patient who discontinued GLPG3970 treatment due to a TEAE had a positive SARS-CoV-2 test result.

AE, adverse event; SARS-CoV-2, severe-acute-respiratory-syndrome-related coronavirus 2; TEAE, treatment-emergent adverse event; UC, ulcerative colitis.

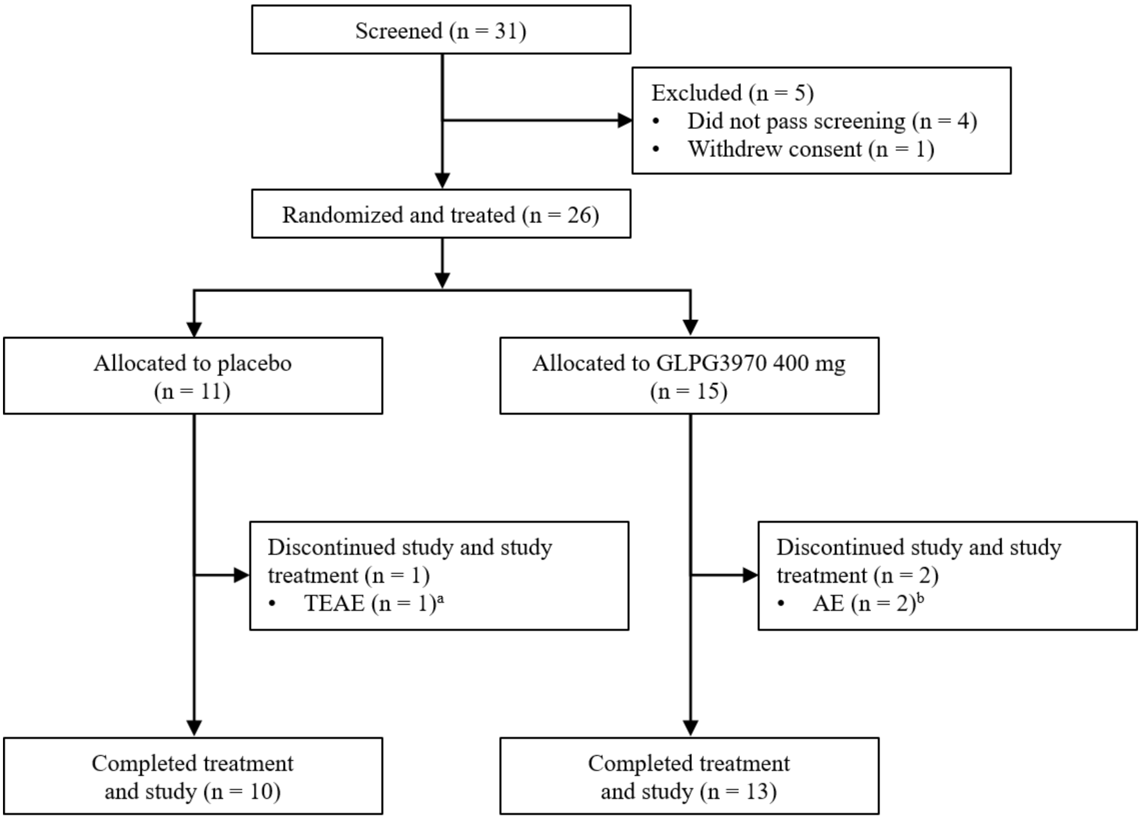

Supplementary Figure 8. Trial profile for PsO

[a]One patient discontinued placebo treatment due to TEAEs of PsO and psoriatic arthropathy. [b]Of patients who discontinued GLPG3970 treatment due to a TEAE, one patient had pruritus, and one patient had a positive SARS-CoV-2 test result.

AE, adverse event; PsO, psoriasis; SARS-CoV-2, severe-acute-respiratory-syndrome-related coronavirus 2; TEAE, treatment-emergent adverse event.

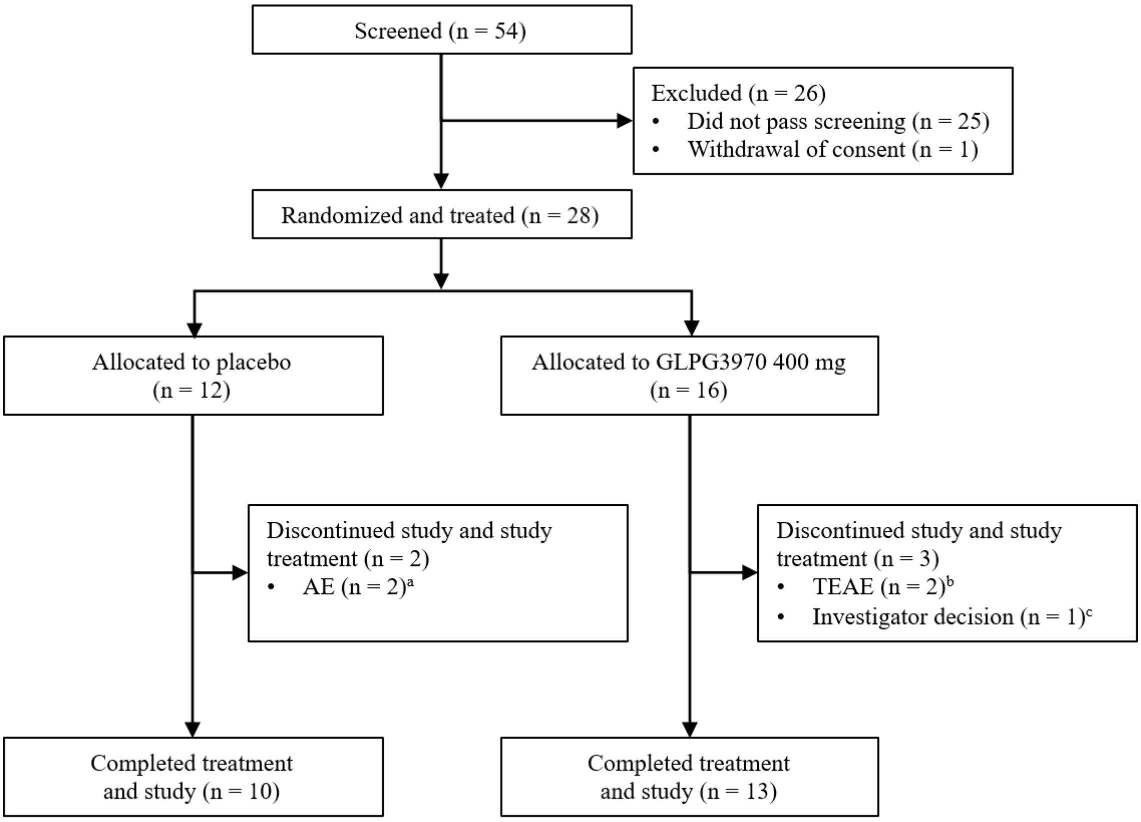

Supplementary Figure 9. Trial profile for RA

[a]Both patients discontinued placebo treatment due to non-treatment-emergent AEs of positive SARS-CoV-2 test result. [b]Of patients who discontinued GLPG3970 treatment due to a TEAE, one patient had increased ALT and one patient had a positive SARS-CoV-2 test result. [c]One patient discontinued GLPG3970 treatment due to an investigator decision as the patient moved abroad.

AE, adverse event; ALT, alanine aminotransferase; RA, rheumatoid arthritis; SARS-CoV-2, severe-acute-respiratory-syndrome-related coronavirus 2; TEAE, treatment-emergent adverse event.

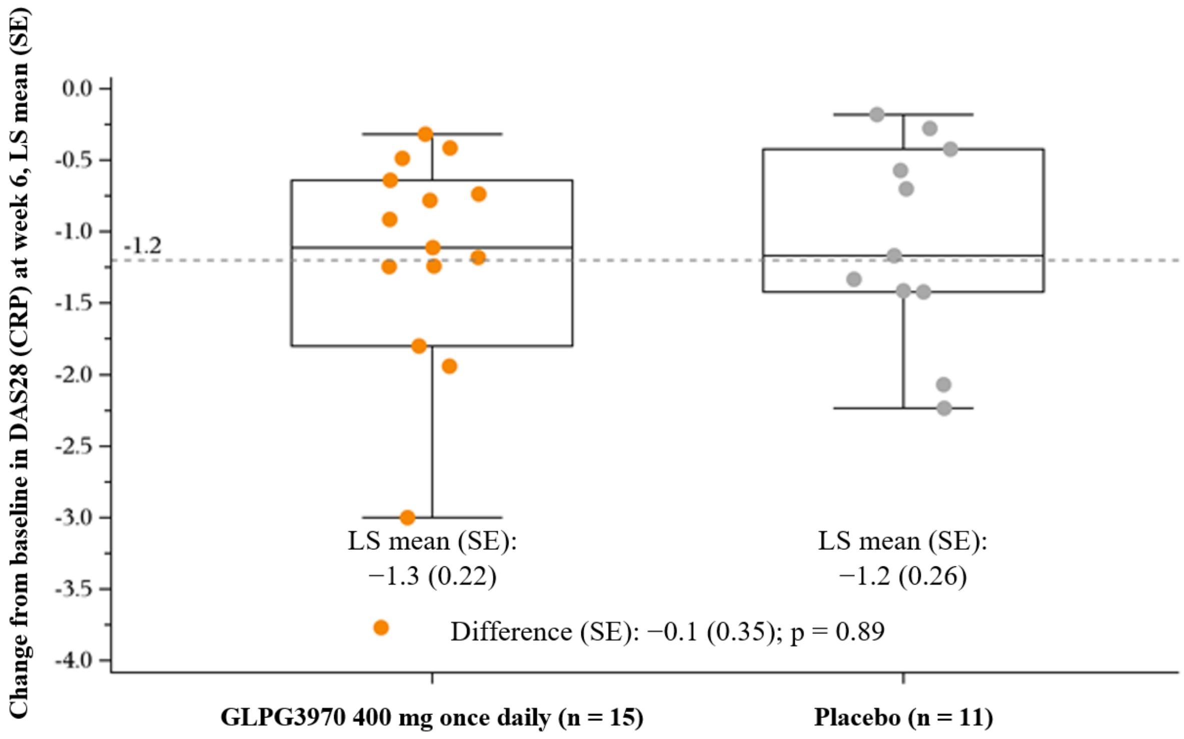

Supplementary Figure 10. Clinical outcomes with GLPG3970 in RA

(A) LS mean change from baseline in DAS28-CRP at week 6 (or last observation post-baseline in case of early study discontinuation, n = 3 patients).

Statistical analyses: LS mean changes were calculated for patients with available data.

CRP, C-reactive protein; DAS28, Disease Activity Score considering 28 joint counts; LS, least-squares; RA, rheumatoid arthritis; SE, standard error.

Supplementary Table 1. Baseline characteristics in UC

	GLPG3970 400 mg<br>N = 21	Placebo<br>N = 10	Total<br>N = 31
Age (years),[a] median (min–max)	36.0 (24–63)	36.0 (32–47)	36.0 (24–63)
BMI (kg/m²),[b,c] median (min–max)	24.9 (20.3–29.9)	24.7 (21.5–29.0)	24.9 (20.3–29.9)
Sex, n (%)			
Male	16 (76.2)	9 (90.0)	25 (80.6)
Female	5 (23.8)	1 (10.0)	6 (19.4)
Race, n (%)			
White	21 (100)	10 (100)	31 (100)
Smoking status, n (%)			
Non-smoker	15 (71.4)	8 (80.0)	23 (74.2)
Ex-smoker	4 (19.0)	1 (10.0)	5 (16.1)
Current smoker	2 (9.5)	1 (10.0)	3 (9.7)
Duration of UC (years), mean (SD, min–max)	7.053<br>(7.160, 0.31–31.87)	6.311<br>(6.075, 0.56–16.11)	6.814<br>(6.736, 0.31–31.87)
MCS total score at baseline, mean (SD, min–max)	8.5 (1.2, 6−10)	8.2 (1.3, 6−10)	8.4 (1.2, 6−10)
MCS-ES at baseline, n (%)			
2: moderate disease (marked erythema, absent vascular pattern, friability, erosions)	8 (38.1)	5 (50.0)	13 (41.9)
3: severe disease (spontaneous bleeding, ulceration)	13 (61.9)	5 (50.0)	18 (58.1)
Fecal calprotectin, mean µg/mg	1460.9 (1523.3, 79 – 5671)	676.0 (528.0, 45 – 1502)	1207.7 (1330.3, 45 – 5671)
hsCRP (mg/L, mean (SD, min-max)	6.40 (6.73, 0.5 – 21.3)	10.94 (23.13, 0.5 -75.7)	7.86 (13.99, 0.5 – 75-7)
RHI at baseline (location: rectum), mean (SD, min–max)	23.1 (8.5, 9−33)	13.9 (9.6, 0−28)	20.2 (9.7, 0−33)
RHI at baseline (location: sigmoid colon), mean (SD, min–max)	17.0 (8.6, 1−33)[b]	8.4 (5.4, 0−17)	14.1 (8.6, 0−33)[d]

[a]Age at the time of screening.

[b]n = 20.

[c]BMI was calculated as weight (kg) divided by (height [m])², rounded to the first decimal.

[d]n = 30.

BMI, body mass index; CRP, C-reactive protein; max, maximum; MCS, Mayo Clinic Score; MCS-ES, Mayo Clinic Score-endoscopic subscore; min, minimum; RHI, Robarts Histologic Index; SD, standard deviation; UC, ulcerative colitis.

Supplementary Table 2. Safety outcomes in UC, PsO, and RA

**UC**			
	**GLPG3970 400 mg N = 21**	**Placebo N = 10**	**Total N = 31**
TEAE, n (%)	11 (52.4)	3 (30.0)	14 (45.2)
Serious TEAE, n (%)	0	0	0
TEAE leading to death, n (%)	0	0	0
TEAE with worst severity, n (%)			
Mild	7 (33.3)	0	7 (22.6)
Moderate	4 (19.0)	2 (20.0)	6 (19.4)
Severe	0	1 (10.0)	1 (3.2)
Life-threatening	0	0	0
Death	0	0	0
Treatment-related TEAE, n (%)	4 (19.0)	1 (10.0)	5 (16.1)
TEAEs leading to study drug discontinuation, n (%)	1 (4.8)[a]	0	1 (3.2)
**PsO**			
	**GLPG3970 350 mg N = 15**	**Placebo N = 11**	**Total N = 26**
TEAE, n (%)	8 (53.3)	4 (36.4)	12 (46.2)
Serious TEAE, n (%)	0	0	0
TEAE leading to death, n (%)	0	0	0
TEAE with worst severity, n (%)			
Mild	3 (20.0)	3 (27.3)	6 (23.1)
Moderate	5 (33.3)	1 (9.1)	6 (23.1)
Severe	0	0	0
Life-threatening	0	0	0
Death	0	0	0
Treatment-related TEAE, n (%)	3 (20.0)	0	3 (11.5)
Treatment temporarily discontinued, n (%)	1 (6.7)	0	1 (3.8)
Treatment permanently discontinued, n (%)	2 (13.3)	1 (9.1)	3 (11.5)
**RA**			
	**GLPG3970 400 mg N = 16**	**Placebo N = 12**	**Total N = 28**
TEAE, n (%)	6 (37.5)	2 (16.7)	8 (28.6)
Serious TEAE, n (%)	0	0	0
TEAE leading to death, n (%)	0	0	0
TEAE with worst severity[a], n (%)			
Mild	4 (25.0)	0	4 (14.3)
Moderate	2 (12.5)	2 (16.7)	4 (14.3)
Severe	0	0	0
Life-threatening	0	0	0
Death	0	0	0
Treatment-related TEAE, n (%)	4 (25.0)	1 (8.3)	5 (17.9)
TEAEs leading to study drug discontinuation, n (%)	2 (12.5)	0	2 (7.1)

Study drug-related is defined as at least possibly drug-related according to the investigator, or a missing drug relatedness.

[a]Only the worst-case within each same preferred term, same patient, and same analysis period is considered (i.e. when the same preferred term for adverse event is reported more than once for the same patient, the patient will be counted only once and will be shown under the worst severity).

PsO, psoriasis; RA, rheumatoid arthritis; TEAE, treatment-emergent adverse event; UC, ulcerative colitis.

Supplementary Table 3. Baseline characteristics in PsO

	**GLPG3970 350 mg N = 15**	**Placebo N = 11**	**Total N = 26**
Age (years),[a] median (min–max)	44.0 (24–62)	36.0 (23–47)	38.5 (23–62)
BMI (kg/m^2),[b] median (min--max)	28.3 (19.7–34.3)	28.3 (21.5–34.6)	28.3 (19.7–34.6)
Race, n (%)			
White	15 (100)	11 (100)	26 (100)
Sex, n (%)			
Male	9 (60.0)	5 (45.5)	14 (53.8)
Female	6 (40.0)	6 (54.5)	12 (46.2)
Duration of PsO (years),[c] median (min–max)	12.8 (5–37)	16.8 (5–33)	13.3 (5–37)
Total PASI score at baseline, median (min–max)	15.6 (12.2–58.0)	16.1 (12.7–30.8)	15.95 (12.2–58.0)
Total body surface area affected (%) at baseline, median (min–max)	28.0 (11–77)	27.0 (10–38)	27.5 (10–77)
sPGA of PsO at baseline, median (min–max)	3.0 (3–4)	3.0 (3–4)	3.0 (3–4)
PtGA of PsO at baseline, median (min–max)	4.0 (3–5)	3.0 (2–5)	4.0 (2–5)
hsCRP result (mg/L) at baseline, median (min–max)	2.350 (0.38–12.91)	1.850 (0.41–12.10)	2.225 (0.38–12.91)

[a]Age at the time of screening.

[b]BMI was calculated as weight (kg) divided by (height [m])^2, rounded to the first decimal.

[c]Duration of PsO (years) was calculated as (date of first screening visit – date of initial PsO diagnosis)/365.25.

BMI, body mass index; hsCRP, high sensitivity C-reactive protein; max, maximum; min, minimum; PASI, Psoriasis Area and Severity Index; PsO, psoriasis; PtGA, patient’s global assessment; sPGA, physician’s static global assessment.

Supplementary Table 4. Baseline characteristics in RA

	**GLPG3970 400 mg N = 16**	**Placebo N = 12**	**Total N = 28**
Age (years),[a] median (min–max)	47.5 (27–61)	39.5 (28–60)	41.0 (27–61)
BMI (kg/m^2),[b] median (min–max)	24.85 (20.0–31.6)	24.80 (22.2–30.5)	24.80 (20.0–31.6)
Sex, n (%)			
Male	5 (31.3)	4 (33.3)	9 (32.1)
Female	11 (68.8)	8 (66.7)	19 (67.9)
Race, n (%)			
White	16 (100)	12 (100)	28 (100)
Duration of RA (years) mean (SD) min–max	7.99 (5.66) 1.8–21.2	7.20 (7.95) 0.7–27.0	7.65 (6.61) 0.7–27.0
hsCRP at baseline (mg/L) mean (SD) min–max	11.86 (12.77) 1.2–54.7	8.76 (5.98) 1.0–18.7	10.53 (10.38) 1.0–54.7
DAS28 (CRP) at baseline mean (SD) min–max	6.13 (0.87) 4.6–7.3	5.68 (0.88) 4.6–6.9	5.93 (0.89) 4.6–7.3
CDAI at baseline mean (SD) min–max	47.2 (15.1) 19–70	40.1 (12.3) 23–60	44.2 (14.2) 19–70

[a]Age at the time of screening.

[b]BMI was calculated as weight (kg) divided by (height [m])^2, rounded to the first decimal.

BMI, body mass index; CDAI, clinical disease activity index; CRP, C-reactive protein; DAS28, disease activity score, considering 28 joint counts; hsCRP, high-sensitivity C-reactive protein; max, maximum; min, minimum; RA, rheumatoid arthritis; SD, standard deviation.
